# A prolific catalyst for dehydrogenation of neat formic acid

**DOI:** 10.1038/ncomms11308

**Published:** 2016-04-14

**Authors:** Jeff Joseph A. Celaje, Zhiyao Lu, Elyse A. Kedzie, Nicholas J. Terrile, Jonathan N. Lo, Travis J. Williams

**Affiliations:** 1 Donald P. and Katherine B. Loker Hydrocarbon Institute and Department of Chemistry, University of Southern California, Los Angeles, California 90089-1661, USA

## Abstract

Formic acid is a promising energy carrier for on-demand hydrogen generation. Because the reverse reaction is also feasible, formic acid is a form of stored hydrogen. Here we present a robust, reusable iridium catalyst that enables hydrogen gas release from neat formic acid. This catalysis works under mild conditions in the presence of air, is highly selective and affords millions of turnovers. While many catalysts exist for both formic acid dehydrogenation and carbon dioxide reduction, solutions to date on hydrogen gas release rely on volatile components that reduce the weight content of stored hydrogen and/or introduce fuel cell poisons. These are avoided here. The catalyst utilizes an interesting chemical mechanism, which is described on the basis of kinetic and synthetic experiments.

## Abstract (web summary)

Formic acid is a promising energy carrier for on-demand hydrogen generation. Here, the authors present a robust, reusable iridium catalyst that enables hydrogen release from neat formic acid under mild conditions and in the presence of air.

## Graphical abstract

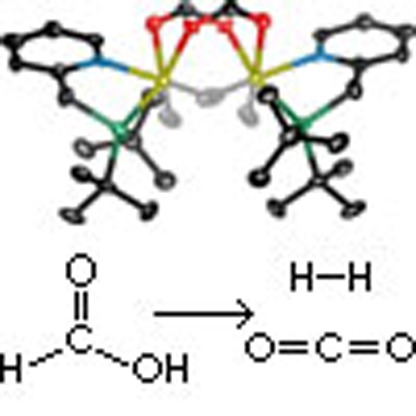

Many strategies for the conversion of solar energy into chemical bonds involve electrocatalytic (or photocatalytic) cleavage of water to form hydrogen and oxygen. The reducing equivalent, H_2_, is thus an energy carrier because it can be re-oxidized, either by combustion to give heat or catalytically in a fuel cell to give electricity. There is a disabling problem with large-scale utilization of hydrogen as a fuel, since it is a gas under ambient conditions, thus limiting its volume-energy density (0.013 MJ l^−1^). As a result, physical methods-based hydrogen storage technologies (compression, cryogenic liquefaction, adsorption) involve low capacity, high costs or safety issues^1^. Therefore, the discovery of highly weight-efficient strategies for on-demand hydrogen release from hydrogen-rich liquids has value. Formic acid (HCO_2_H, FA, 7.5 MJ l^−1^) is a hydrogen carrier, owing to its ability to release hydrogen under mild conditions with only CO_2_ as a by-product^1^,^2^,^3^,^4^,^5^,^6^, which can then be recycled, in principle, to give a carbon-neutral fuel cycle^7^,^8^,^9^.

To date, many efficient heterogeneous^10^,^11^,^12^,^13^,^14^,^15^,^16^,^17^,^18^,^19^,^20^,^21^,^22^ and homogeneous^9^,^23^,^24^,^25^,^26^,^27^,^28^,^29^,^30^,^31^,^32^,^33^,^34^,^35^,^36^,^37^,^38^ catalysts for FA dehydrogenation have been developed. Heterogeneous catalysts have advantages of separability and reusability^11^, while homogeneous catalysts are generally more efficient. The best turnover numbers (TONs) achieved in homogeneous catalysis are (1) >1M, by a catalyst system composed of [RuCl_2_(benzene)]_2_, the ligand diphenylphosphinoethane and a FA/Et_3_N adduct as substrate developed by Boddien *et al*.^9^ and (2) 983,642, by a system composed of an iron pincer complex and LiBF_4_ developed by Bielinski *et al*.^35^. The highest turnover frequency achieved is 228,000 h^−1^ by an iridium catalyst developed by Hull *et al*.^27^. Supplementary Table 1 has a more complete comparison of homogeneous catalysts for this reaction. In heterogeneous catalysis, the highest TOF achieved is 7,256 h^−1^, by palladium nanoparticles immobilized on carbon nanospheres developed by Zhu *et al*.^22^. Also, homogeneous catalysts generally are more selective, producing less carbon monoxide, a common byproduct of FA dehydrogenation. This is essential, because CO is a fuel cell catalyst poison. Still, no known system is stable and reactive through multiple uses, air and water tolerant, selective against CO formation, and functions in neat FA liquid. Each of these is critical to achieving a usable hydrogen generation system based on FA. Herein we report an iridium-based catalytic system that meets all of these criteria.

## Results

### Reactivity in FA dehydrogenation

**Figure 1:**
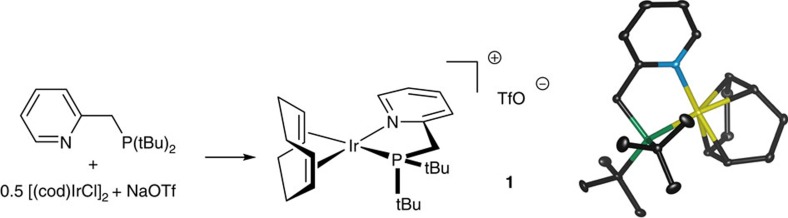
Synthesis and structure of catalyst precursor cation 1. Elipsoids are drawn at the 50% probability level.

Complex **1**, which is easily prepared from known materials (Fig. 1), decomposes FA (500 μl, 12.7 mmol) with NaO_2_CH co-catalyst (5 mol%) at 50 p.p.m. loading and 90 °C, resulting in the production of 386 ml of gas (62% conversion; TON=12,530) after 13 h. The mass balance of FA condenses as a liquid in the reactor out of reach of the catalyst (*vide infra*). The rate of the reaction is constant through ca. 20% of conversion before it accelerates as FA disappears (Supplementary Fig. 1). At the end of the reaction, a pale orange solid (the catalyst system: an iridium complex and sodium formate) remains at the bottom of the reaction vessel. Recharging the reaction flask with FA and reheating to 90 °C results in continued H_2_ production without any catalyst regeneration.

The reaction requires base as co-catalyst, but the source of the base is not specific: the reaction rates are similar when 5 mol% NaO_2_CH, KO_2_CH, KOH, NaOH, LiOH, or nBu_4_NOH or 2.5 mol% of Na_2_CO_3_ or K_2_CO_3_ is used (Supplementary Table 2). Any of these is converted rapidly to the corresponding formate, which comprises the bulk of the catalytic material and gives it its pale color. Moreover, water does not affect the rate of dehydrogenation significantly—the double logarithmic plot of water concentration versus rate of FA dehydrogenation yields a slope of 0.11(5) (Supplementary Fig. 2).

The catalysts are air stable. Although dehydrogenation is slower when the catalysts are prepared in air, the system remains active, even when the solution is allowed to sit on the bench top for 2 weeks before dehydrogenation rates are measured (Supplementary Table 3). Under these conditions, the catalysts can be re-loaded in an air atmosphere and re-used repeatedly. For example, a reaction flask containing iridium **1** (6.1 mg, 8.9 μmol) and NaO_2_CH (185 mg, 2.72 mmol) was charged with FA through 50 cycles (Supplementary Table 4). In this experiment 28.85 l of gas was produced from 25 ml of FA, corresponding to a TON of 66,403 and 89% conversion. Note that these values exclude the FA liquid that condenses in the flask out of reach of the catalyst in each run and accounts for all mechanical leaks or engineering challenges in the laboratory-scale set-up. In a particular, representative single run, we converted FA (2 ml) to gaseous products in 97% conversion with 140 p.p.m. **1** and 280 p.p.m. sodium formate. Over the course of 50 loadings, we measured the initial rates and maximum turnover frequencies during certain runs (Table 1). Interestingly, these increased over the course of 10 cycles before slowing over time.

The iridium catalyst delivers very high TONs at low loading with repeated re-use. For example, we prepared in the drybox a reaction flask containing **1** (90 μg, 0.13 μmol) and NaO_2_CH (184 mg, 2.65 mmol) and repeatedly charged it with FA, which was decomposed until a pale yellow solid remained at the bottom of the flask. After 40 cycles over a period of four months, 13.71 l of gas was produced, which corresponds to a TON of 2.16 million (Supplementary Table 5). Although unoptimized, this is the best TON for a FA dehydrogenation catalyst known to date to our knowledge. Under these conditions, the maximum TOF was measured to be 3.7 s^−1^.

The reaction is operationally simple. The catalytic materials are weighed out in a reactor, which is attached to a vent line for the gaseous products. Liquid FA is added, and the reaction is initiated by heating. Upon completion, the catalyst system remains as a pale-coloured precipitate at the bottom of the vessel for re-use.

**Figure 2:**
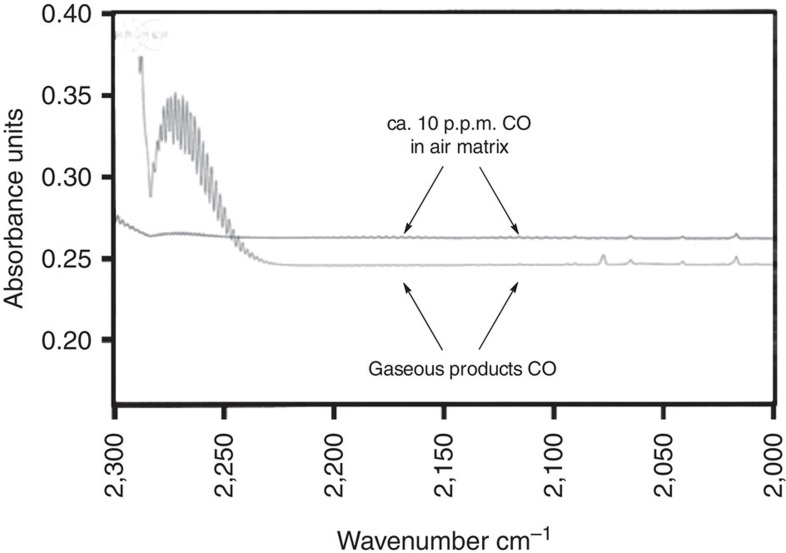
Gas eluent stream infrared spectrum. The figure shows that compared to a prepared sample with 10 p.p.m. CO in air, the gaseous products from dehydrogenation of neat formic acid saturated in sodium formate contain <10 p.p.m. CO.

To be useful in fuel cells, FA decomposition must be selective for H_2_ and CO_2_ over H_2_O and CO, because CO is a poison for polymer electrolyte membrane (PEM) fuel cell catalysts such as platinum. The composition of gas produced from our conditions was determined by gas chromatography, which showed only H_2_ and CO_2_ (1:1 ratio) and no detectable CO (<1 part per thousand) (Supplementary Fig. 3). However, further analysis of the product gas by infrared spectroscopy revealed that when the reaction is conducted using neat FA, CO is observed at a concentration near the detection limit of the gas chromatography (Supplementary Fig. 4). It is known that neat FA decomposes in the presence of concentrated acid^39^ or at high temperatures to form H_2_O and CO^40^,^41^. We therefore hypothesized that much of the CO produced in our reaction conditions may be formed by thermal, uncatalysed decomposition pathways. Thus, we performed the dehydrogenation in the presence of a portion of water (10 v%), and observed that under these conditions the CO in the bulk gaseous products is <10 p.p.m. by infrared spectroscopy (Supplementary Fig. 5). Moreover, we hypothesized that the thermal decomposition of neat FA might be suppressed by running the reaction using higher sodium formate loading. Indeed, heating 1 ml of neat FA in 26 mg of the iridium precatalyst and 900 mg of sodium formate (50 mol%) to 90 °C for 2 h yields a product mixture with less than 10 p.p.m. CO (Fig. 2). A similarly low level of CO is observed when dehydrogenation is performed at 70 °C for 6 h (Supplementary Fig. 6). While these strategies for CO minimization are known in the FA literature, this collection of demonstrations enables practitioners to select the level of humidity and CO content in the reaction's gas eluent stream simply by adjusting the water and base loading in the FA supply. The optimum of these parameters might be different for any particular fuel cell application, but the reaction affords flexibility to adjust them.

We find that the active catalyst is homogeneous on the basis of physical appearance, clean kinetics, tolerance of liquid mercury and proportional inhibition by phenanthroline^42^ (Supplementary Methods). Accordingly, the system exhibits the reactivity and selectivity advantages of homogeneous catalysis. Nonetheless, because the catalytic materials are deposited cleanly in the reactor at the end of the reaction, the system enjoys many of the catalyst separability and reusability benefits of heterogeneous conditions.

### Mechanistic studies

**Figure 3:**
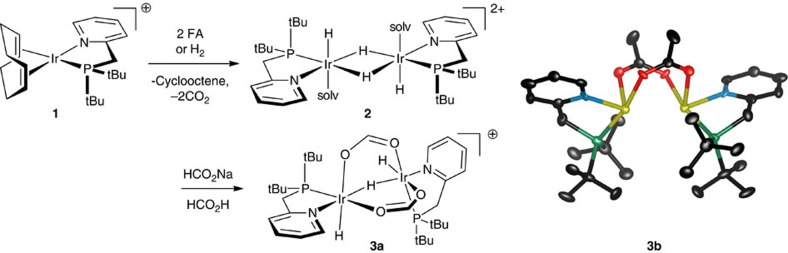
Catalyst initiation and molecular structure of active catalyst homologue 3b. **3b**={[(tBu_2_PCH_2_(2-py))Ir(H)]_2_(μ^2^-H)(μ^2^-κ,κ′-O_2_CCH_3_)_2_}^+^. Hydrogen atoms omitted. Ellipsoids are drawn at the 50% probability level. counterion, trifluoromethanesulfonate; solv, solvent.

**Figure 4:**
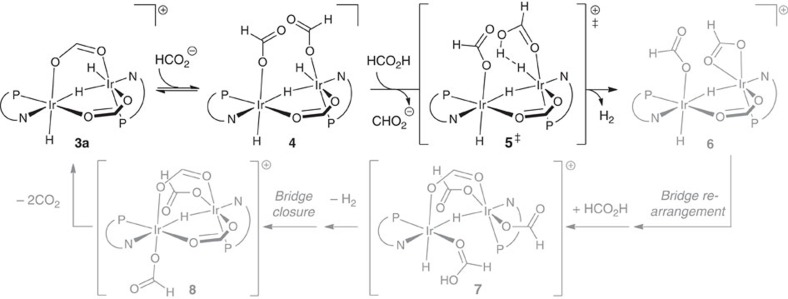
Proposed mechanism of catalysis. Counterion, trifluoromethanesulfonate. ‡Refers to a proposed transition state. The P–N ligand is 2-((di-tbutylphosphino)methyl)pyridine.

Equally remarkable as the reactivity of this new catalytic system is the unique, two-metal mechanism through which it operates. We used three approaches to gain insight into this mechanism: stoichiometric model reactions, reaction kinetics and isotope labelling studies. Figures 3 and 4 present a sketch of a possible mechanism for our system.

Species **1** is a catalyst precursor from which an active catalyst is generated. To determine the nature of this active species, we conducted stoichiometric reactions of **1** (Fig. 3). Species **1** loses its cyclooctadiene ligand as cyclooctene in a solution of either H_2_ or buffered FA and dimerizes to form **2**. Complex **2** has analogy to {[(P-N)Ir(CH_2_Cl_2_)(H)]_2_(μ^2^-H)_2_}^2+^ characterized by Pfaltz^43^ (P-N=SimplePHOX). In buffered FA conditions, **2** is then converted to a formate-bridged species **3a**. While this species is observable by NMR, it is not amenable to isolation in our hands. By contrast, its acetate homologue (**3b**) yielded to crystallization, which enabled determination of its structure (Fig. 3). Species **3a** is relevant in catalysis: we observe it by NMR as the minor form of the working catalyst. We see a second, major resting species by NMR, which has a spectrum consistent with structure **4**, featuring three differentiated metal hydride groups. The NMR spectra of intermediates are shown in Supplementary Figs 7–11, which are further analysed in Supplementary Discussion.

Kinetic isotope effect data indicate that both the C–H and O–H groups of FA are involved in (or before) the rate-determining transition state. Table 2 summarizes the reaction rates for four selectively labelled FA isotopologues. The combined isotope effect (*k*_CHOH_/*k*_CDOD_=6.5(2)) is comparable to the product of the average separate C–H and O–H isotope effects (6.5(4)). This is consistent with a mechanism by which bonds to proton and hydride are transformed in a single kinetically relevant step. Hydrogen loss from **4** involves protonation of an iridium hydride (which comes from FA's C–H group) by a FA group. Further, we observe that in a sample of FA–(*O*)–*d*_1_, NMR reveals H-D gas as the catalytic product (Supplementary Fig. 12 and Supplementary Discussion). A small portion of H_2_ is formed in the process of catalyst initiation, but none is detected during catalysis. This indicates that there is separation of proton and hydride groups throughout the mechanism and refutes the possibility of an iridium dihydride species in the mechanism, because such a species would be likely to enable proton/hydride scrambling via reversible reductive elimination of dihydrogen. This observation also shows that the reaction is irreversible at ambient pressure, so we assign the isotope effects as kinetic.

Eyring analysis reveals activation parameters of ΔH^‡^=+29.0(3) kcal mol^−1^ and ΔS^‡^=+16(1) eu (ΔH^‡^=121(12) kJ mol^−1^; ΔS^‡^=+67(4) J mol^−1^ K^−1^) (Supplementary Fig. 13). This strongly favourable entropy of activation is consistent with the release of at least one gaseous product in the rate-determining transition state. We expect that this is H_2_ release in the conversion of **4** to **6** because of the strong isotope effects.

The observed rate law for FA dehydrogenation has rate∼[Ir]^1^[base]^0.5^[FA]^−1^, which is based on the slopes of double logarithmic plots (Supplementary Figs 14–18) recorded both in neat FA and dilute in tetraglyme solution (Table 3). This rate law requires that two sites of the catalyst are activated by a single equivalent of formate, thus causing half-order dependence on base. We propose a possible catalytic cycle in Fig. 4. After the first equivalent of H_2_ is lost in the conversion of **4** to **6**, a second equivalent forms from the iridium hydride on the complementary metal centre. We propose that the latter is more rapid than the former, and that the single equivalent of formate enables both by opening a formate bridge in dimer **3a**. The rate law also has [Ir] first order, which indicates a dimeric iridium species that does not dissociate once formed. Inverse order in [FA] implies inhibition, but the origins of this inhibition are unclear. Acid is known to favour closure of carboxylate bridges in ruthenium species similar to ours^44^, which enables several opportunities for FA inhibition in our mechanism. Moreover, FA has potential roles in the conversion of **4** and as solvent. We are currently studying this complex system of interactions.

## Discussion

We show here a new catalytic system for the repeated conversion of FA to CO_2_ and hydrogen. This has translation potential because it is the first known homogeneous system, to the best of our knowledge, to operate in neat FA, thus enabling far greater weight content of H_2_ release than any other known catalyst for FA dehydrogenation. Moreover, it is the highest turnover system, because, in part, it can be re-used directly with FA substrate that is not rigorously purified or dried. We further propose a mechanism to account for kinetic, thermochemical, stoichiometric and labelling data that we have collected for the catalytic reaction. More detailed mechanistic studies, including computational investigation and ligand variation, are currently under way.

## Methods

### Synthesis of complex 1

General experimental information and characterization details can be found in the Supplementary Methods. In the drybox under nitrogen, 2-((di-t-butylphosphino)methyl)pyridine^45^ (105.3 mg, 0.44 mmol) was dissolved in a dry vial in 5 ml of dry dichloromethane. In another vial containing a Teflon stir bar, chloro(1,5-cyclooctadiene)iridium(I) dimer (149.0 mg, 0.22 mmol) and sodium triflate (130 mg, 0.75 mmol) were suspended in 10 ml of dry dichloromethane. The suspension was stirred vigorously and then the phosphinopyridine solution was added slowly dropwise. The phosphinopyridine vial was rinsed with 5 ml of dichloromethane and added to the stirred suspension. After stirring for 1 h, the solution was filtered to remove the sodium chloride byproduct and the excess sodium triflate. The solvent was evaporated under reduced pressure to yield an orange glassy solid. A 5:1 mixture of dry hexanes/ethyl ether (10 ml) was added to the residue and then triturated by sonication. The hexane was decanted and the residue washed with an additional 10 ml of hexanes/ethyl ether. The pure iridium complex was dried under reduced pressure to give an orange solid (235 mg, 77.3%). Recrystallization from dichloromethane and toluene produced crystals suitable for X-ray crystallography (Supplementary Data 1). NMR spectra of complex **1** are shown in Supplementary Figs 19–22.

### Dehydrogenation procedures

The dehydrogenation of FA can generally be performed by preparing a stock solution of the catalysts. In the drybox, formate and the iridium precatalyst are dissolved in either FA or tetraglyme solvent. The resulting orange solution slowly turns pale yellow over the course of ca. 1 h. The solution is allowed to sit for several hours or overnight before the catalyst is used for dehydrogenation reactions.

### Reactions followed to completion

In the drybox, a 0.5-ml aliquot of a stock solution is transferred into a 5-ml high-pressure reaction flask possessing a side arm and a large-bore plug valve. The flask is then taken out of the drybox and connected to a vent line leading to a gas burette filled with oil (a eudiometer). To follow the reaction to completion, a 1,000-ml gas burette is used. The reaction flask is heated to 90 °C in an oil bath for ca. 15 min before opening the valve. The volume of gas produced over time is recorded. Some portion of the liquid FA vapourizes and re-condenses in the head space of the flask and gas evacuation tube, which prevents complete conversion.

### Accurate measurements of initial rates

In the drybox, a 0.5-ml aliquot of a stock solution is transferred into a 5-ml reaction flask possessing a large-bore plug valve and a side arm. This flask is taken out of the drybox and the sidearm is connected to a three-way valve, which is connected to a nitrogen line and a 50-ml gas burette. The tubing and gas burette are purged with nitrogen for ca. 15 min. The reaction flask is then heated in an oil bath to 86 °C. Because the oil bath temperature increases after initial heating, the mixture is heated for ca. 15 min before readings are taken to allow the temperature to equilibrate. The volume of gas formed over time was then recorded. The initial rate of FA decomposition (average of two runs) was obtained from a plot of moles of FA decomposed versus time (20 data points were obtained in each experiment). A sample plot is shown in Supplementary Fig. 23.

### Method of initial rates

Because the rate of FA dehydrogenation is constant at the beginning of the reaction (Supplementary Fig. 1), we used the method of initial rates to obtain kinetics data to compare directly the dehydrogenation rates when different bases are utilized, to construct an Eyring plot, to measure kinetic isotope effects, and to determine the effect of water and of different poisons (that is, mercury and phenanthroline). Also, this method was used to study the reaction order in the iridium catalyst, the base and FA to determine the rate law. See the Supplementary Information for full details.

### Obtaining the X-ray structure of the catalyst homolog **3b**

In the drybox under nitrogen, complex **1** (10 mg, 14.9 μmol) was dissolved in 0.6 ml dichloromethane-*d*_2_ in a J-Young NMR tube. Dry acetic acid (8.6 μl, 149 μmol) was also added to this solution. The tube was then degassed, put under 1 atm head pressure of H_2_ gas, and shaken. After ca. 5 min, a ^1^H NMR spectrum of the crude reaction mixture was obtained, which confirmed the formation of **2**. The solution was then poured into a dry 1-dram vial. Hexane was carefully layered on top of this dichloromethane solution, and the vial was left in a desiccator for 1 week. A crystal suitable for X-ray diffraction was isolated from the vial (Supplementary Data 2). Although the crystal of **3b** is stable for days, the pure crystal of **3b** re-dissolved in dichloromethane-*d*_2_ appears to be in equilibrium with **2** and potentially any other form of the iridium complex. NMR spectra of complex **3b** are shown in Supplementary Figs 24–27.

## Additional information

**Accession codes:** The X-ray crystallographic coordinates for the structures reported in this Article have been deposited in the Cambridge Crystallographic Data Centre (CCDC) under deposition numbers CDCC #1415049 (**1**) and #1415050 (**3b**).

**How to cite this article:** Celaje, J. J. A. *et al*. A prolific catalyst for dehydrogenation of neat formic acid. *Nat. Commun.* 7:11308 doi: 10.1038/ncomms11308 (2016).

## Supplementary Material

Supplementary Information Supplementary Figures 1-27, Supplementary Tables 1-5, Supplementary Discussion, Supplementary Methods and Supplementary References

Supplementary Data 1 Crystallographic information for 1

Supplementary Data 2 Crystallographic information for 3b

## Figures and Tables

**Table 1:** Catalyst performance over iterative uses.

**Entry**	**Loading**	**Initial rate (μmol s**^**−1**^**)**	**Maximum turnover frequency (h**^**−1**^**)**
1	1st	0.52	1,378
2	10th	2.35	3,032
3	20th	2.77	2,756
4	30th	2.82	2,618
5	40th	2.51	2,205
6	50th	1.46	1,519

**Table 2:** Kinetic isotope effect data.

**Compound**	***k***_**rel**_	**KIE (observed)**	
HCO_2_H	6.5 (2)	*k*_CHOH_/*k*_CHOD_	1.8 (3)
HCO_2_D	3.6 (2)	*k*_CDOH_/*k*_CDOD_	1.65 (3)
DCO_2_H	1.6 (2)	*k*_CHOH_/*k*_CDOH_	3.9 (2)
DCO_2_D	1.00 (2)	*k*_CHOD_/*k*_CDOD_	3.6 (2)
		*k*_CHOH_/*k*_CDOD_	6.5 (2)

Conditions are 50 p.p.m. **1**, 5 mol% base, 86 °C.

**Table 3:** Reaction kinetics.

	Neat*	Solution*†
[Ir]	0.95(3)‡	0.96(4)||
[base]	0.64(5)¶	0.44(2)#
[FA]	-	−0.94(9)**

^*^Data were collected at 86 °C as an average of two runs.

^†^Tetraglyme was used as solvent. Base was delivered as (*n*-Bu)_4_NOH to generate soluble (*n*-Bu)_4_N(O_2_CH).

^‡^Data were collected using 0.63 M [NaO_2_CH] (2.5 mol%) and [Ir] concentrations of 0.63, 1.86, 2.59, 3.25 and 4.41 mM.

^||^Data were collected using 13.2 mM [(*n*-Bu)_4_N(O_2_CH)] (5 mol%) and [Ir] concentrations of 0.066, 0.13, 0.20, 0.26 and 0.33 mM.

^¶^Data were collected using 0.66 mM [Ir] and [NaO_2_CH] concentrations of 0.26, 0.53, 1.06, 1.59, 2.11 and 2.65 M.

^#^Data were collected using 0.066 mM [Ir] and [(*n*-Bu)_4_N(O_2_CH)] concentrations of 13.2, 26.4, 39.6, 52.8 and 66.0 mM.

^**^Data were collected using 0.026 [Ir], 13.2 mM [(*n*-Bu)_4_N(O_2_CH)] and [FA] concentrations of 265, 331, 398, 530 and 662 mM.
