# Supplementary material for: A prolific catalyst for dehydrogenation of neat formic acid
Source: Nat Commun. 2016 Apr 14;7:11308. doi: 10.1038/ncomms11308 (PMC4834634; doi:10.1038/ncomms11308)
Supplement: Supplementary Information — Supplementary Figures 1-27, Supplementary Tables 1-5, Supplementary Discussion, Supplementary Methods and Supplementary References [file ncomms11308-s1.pdf]

Supplementary Figures

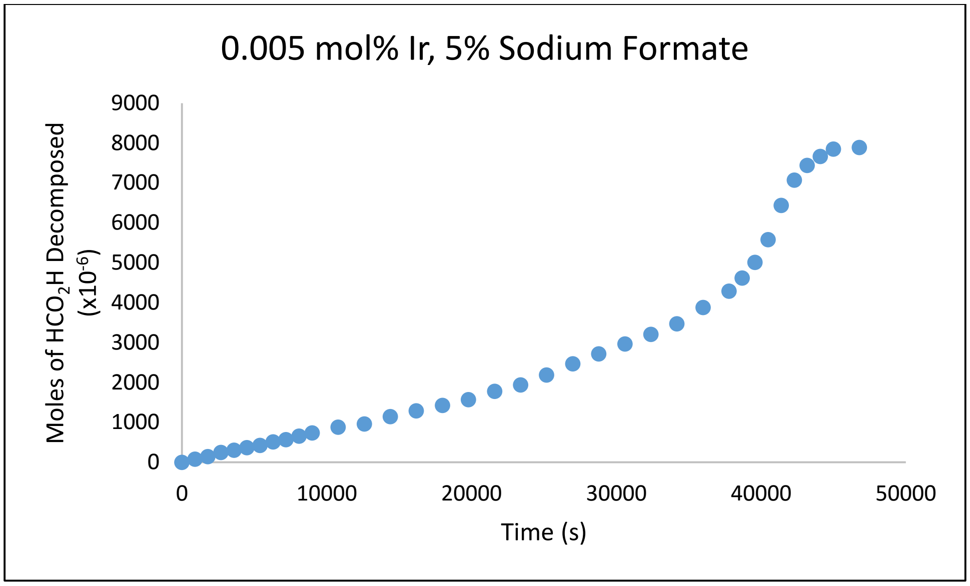

Supplementary Figure 1 | Plot of moles of formic acid decomposed versus time. The dehydrogenation of a formic acid solution which was initially 0.005 mol% (50 ppm) in iridium atoms and 5 mol% in sodium formate was followed. The volume of gas produced was recorded and directly converted to the number of moles of formic acid decomposed. One mole of formic acid produces 48.91 L of gas (24.49 L of H_2 and 24.42 L of CO_2)[1,2]. A plot of moles of formic acid decomposed versus time was constructed and the rate of formic acid decomposition at any point over the course of the reaction can be measured. Because the rate is constant at the beginning of the reaction, we can use the method of initial rates to study the reaction order of the three reagents involved in the reaction: the Ir catalyst, the base, and formic acid. We also used the method of initial rates to directly compare the dehydrogenation rates when different bases are utilized, to construct an Eyring plot, to measure kinetic isotope effects, and to determine the effect of water and of different poisons (i.e. mercury and phenanthroline).

A.

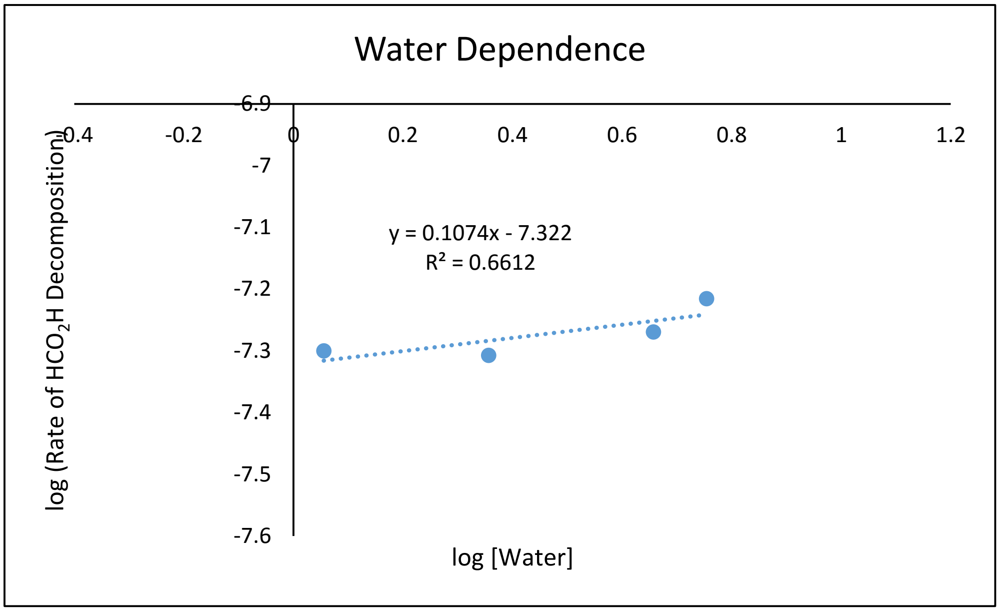

B.

Water added (µL)	[Water] (M)	[Ir] (mM)	[NaO_2CH] (M)	[FA] (M)	Rate (10^{-8} mol s^{-1}) [a]
10	1.14	1.14	1.14	24.96	5.0(3)
20	2.28	1.14	1.14	24.48	4.9(8)
40	4.54	1.14	1.14	23.52	5.4(6)
50	5.68	1.14	1.14	23.04	6.1(3)

[a] Obtained at 86 °C; average of two runs, error is one standard deviation.

Supplementary Figure 2 | A. Plot of log (calculated rate of formic acid decomposition) versus log [water] (formic acid solvent). B. Data used to obtain log/log plot for water dependence. In the drybox, a formic acid stock solution that was 0.005 mol% (50 ppm) in iridium precatalyst **1** and 5 mol% in sodium formate was prepared by dissolving 1.8 mg (2.6 µmol) of **1** and 180 mg (2.64 mmol) of sodium formate in 2.0 mL of formic acid. 0.5 mL of this stock solution was transferred into a 5 mL reaction flask possessing a large bore plug valve and a side arm. Water is added according to the amounts shown in the table above. Additional formic acid is added accordingly to make a 0.55 mL solution, which was used to measure the rates of formic acid decomposition using Procedure 2 (see Methods). A log/log plot of the rate of formic acid decomposition versus the water concentration yields a slope of 0.11(5). Thus, water does not inhibit the rate of dehydrogenation.

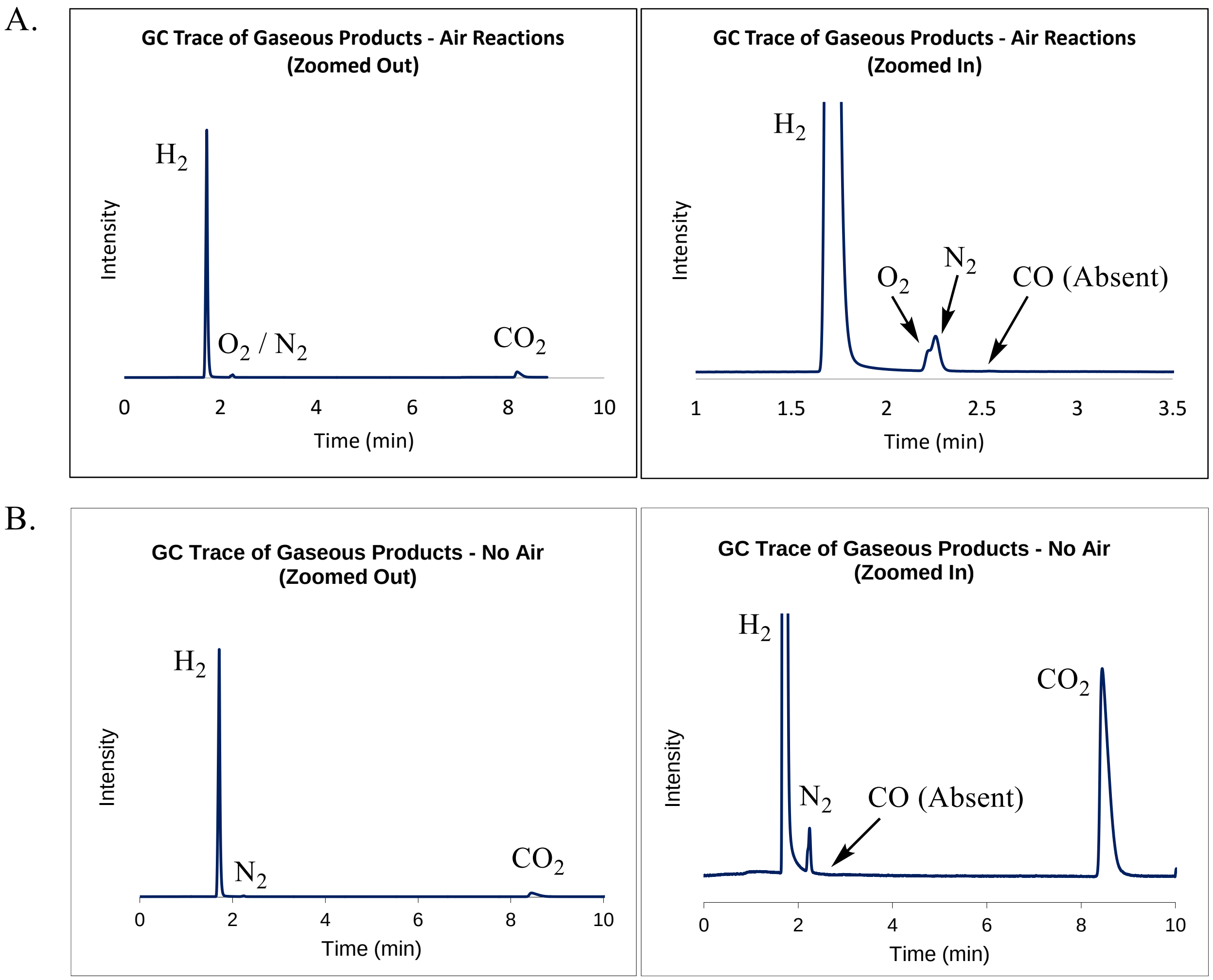

Supplementary Figure 3 | A. GC trace of gaseous products from a dehydrogenation reaction performed in the presence of air. B. GC trace of gaseous products from a dehydrogenation reaction performed in the absence of air. Supplementary figure 3A was obtained using the following procedure. To a Schlenk test tube containing the iridium precatalyst (5.8 mg, 8.4 µmol) and NaO_2CH (182.6 mg, 2.7 mmol) was added, in air, 0.5 mL of formic acid. The Schlenk test tube was connected to a three-way valve which was connected to a balloon. The reaction flask was heated to 90 °C. The gaseous products were collected in the balloon until almost all formic acid was decomposed and their composition analyzed by gas chromatography using a thermal conductivity detector. Three runs were performed. A 1:1 mole ratio of H_2 and CO_2 was detected along with small amounts of oxygen and nitrogen (figure A above, carbon monoxide was not detected; thus, the carbon monoxide level is < 0.099 v/v %). Figure B was obtained using a similar procedure with glove box and Schlenk techniques to exclude air.

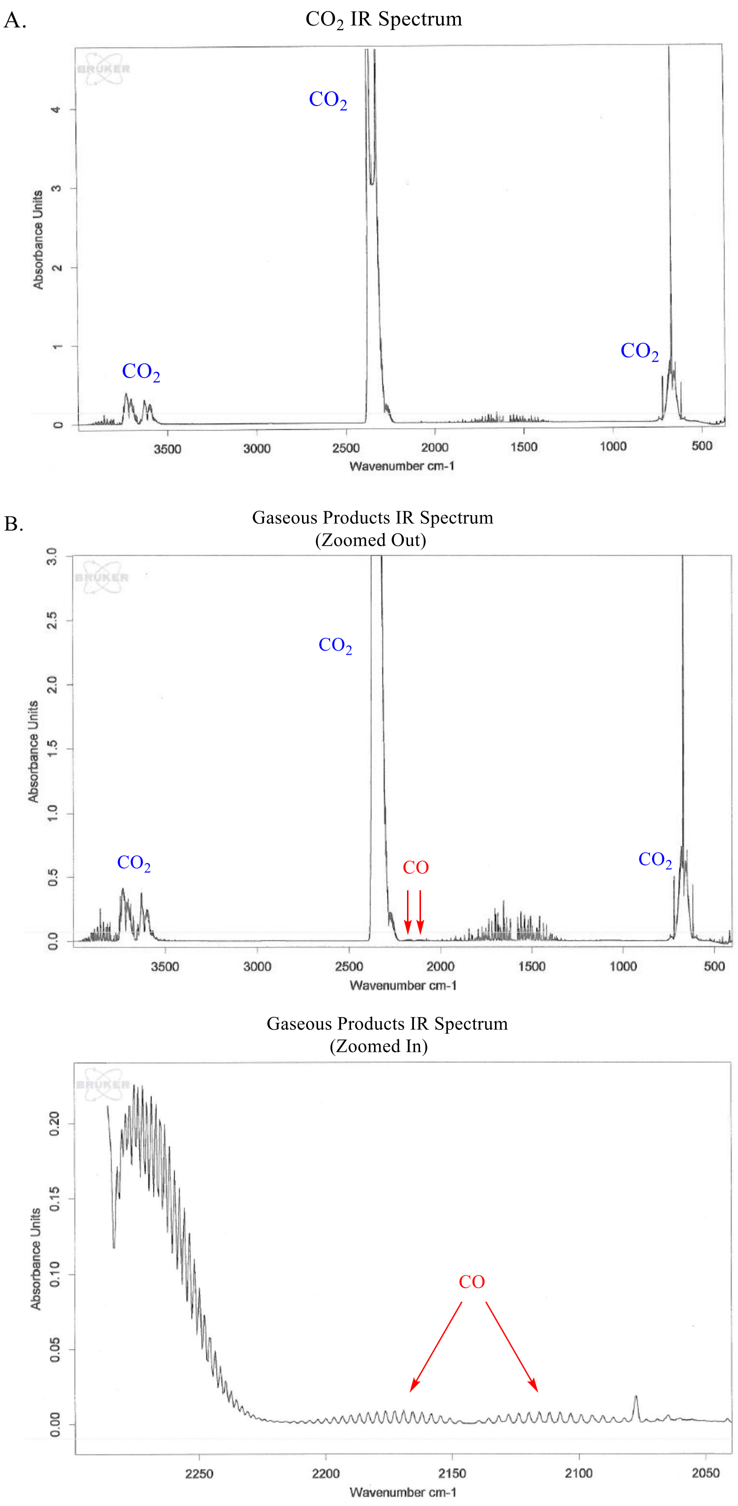

Supplementary Figure 4 | A. IR spectrum of commercial CO_2. B. IR spectrum of gaseous products from neat formic acid. A 50 mL Schlenk tube was charged with iridium precatalyst **1** (6.1 mg, 8.9 μmol) and sodium formate (184.0 mg, 2.7 mmol), which were dissolved, in air, in 1.0

mL formic acid. The Schlenk test tube was connected to a three-way valve, which was connected to a 2 L gas burette. The gas burette was fitted with a vent line connected to a three-way valve, which allows for direct sampling of the gaseous products for IR analysis. The reaction flask was heated to 90 °C. Gaseous products were collected until 1.1 L of gas was produced (ca. 5 h). An aliquot for IR analysis was transferred using a syringe into a Harrick temperature controlled gas cell (10 cm path length, 17 mL cell volume), which had been evacuated under reduced pressure. IR data (1 cm^{-1} resolution) was then obtained on a Bruker Vertex 80v FT-IR instrument equipped with a DTGS detector. Unlike a spectrum of commercial gaseous CO_2 (A), CO can be observed in the IR spectrum of the gaseous reaction products (B). Using a calibration curve of different CO concentrations in air matrix, we estimated the CO concentration to be ca. 500 ppm. We also performed dehydrogenation of 5 mL of neat formic using the same catalyst loading over a period of 60 h. In this case, the CO concentration was estimated to be ca. 2000 ppm.

A.

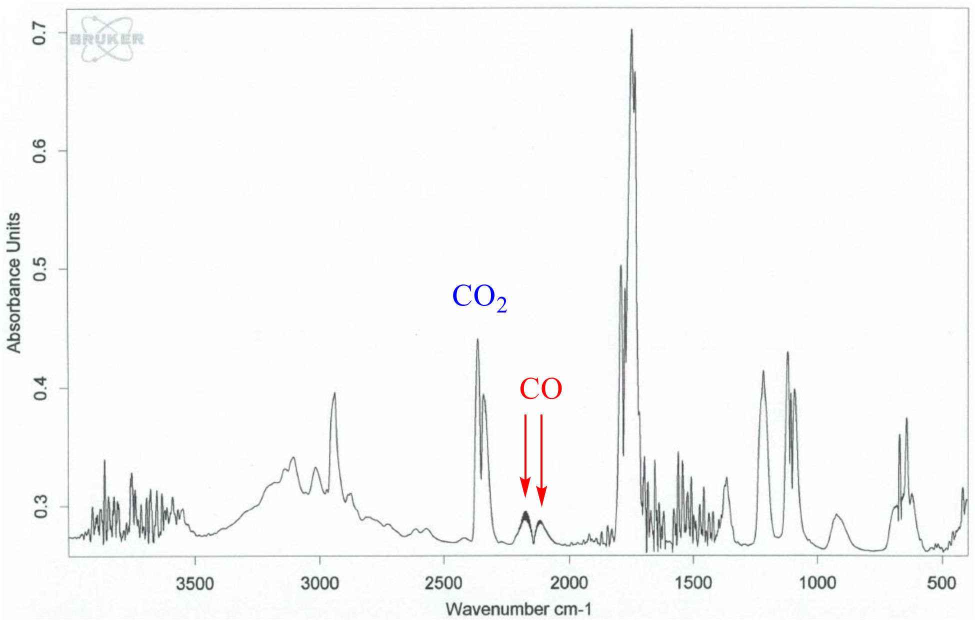

Supplementary Figure 5 | A. IR spectrum of the headspace of a reaction flask containing neat formic acid heated to 90 °C for 2 h. It is known that neat formic acid decomposes in the presence of concentrated acid or at high temperatures to form H_2O and CO[3,4,5]. We therefore hypothesized that much of the CO produced in our reaction conditions may be forming because of thermal, uncatalyzed decomposition of neat formic acid. To test for this situation, we dissolved 180 mg of sodium formate in 1 mL of formic acid and heated this solution in a sealed 100 mL pear flask for 2 h at 90 °C. The solution was then allowed to cool to room temperature and a sample of the headspace gases was analyzed by IR. Indeed, even after only 2 h of heating, copious amounts of CO (which was estimated to >5000 ppm) had formed as seen in the IR spectrum above.

B. Comparison of Gaseous Products CO to ca. 10 ppm CO
(Zoomed Out)

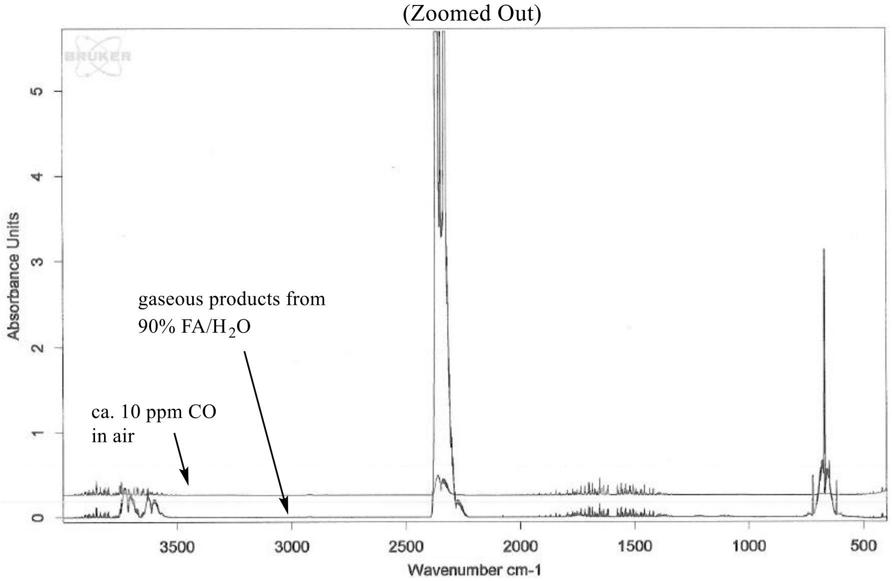

Comparison of Gaseous Products CO to ca. 10 ppm CO
(Zoomed In)

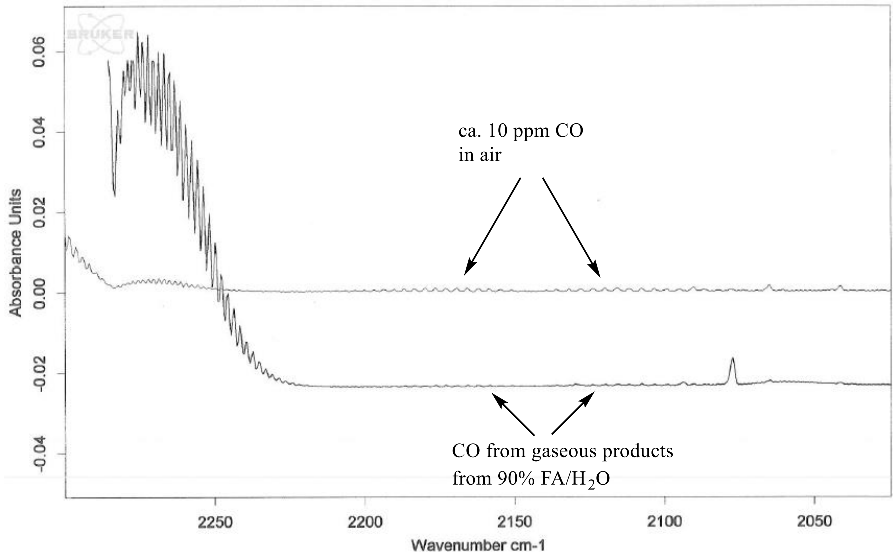

Supplementary Figure 5 | B. Comparison of IR spectra of gaseous products from 90 v/v% FA/H_2O and ca. 10 ppm CO in air matrix. Because it is known that neat formic acid thermally decomposes to produce H_2O and CO, and that the presence of water decreases the rate of this decomposition[3,4,5], we reasoned that we might be able to suppress CO production by using 10 vol% H_2O/FA instead of neat formic acid as the dehydrogenation medium. Thus, a 50 mL Schlenk tube was charged with iridium precatalyst **1** (30.2 mg, 44.0 μmol) and sodium formate (181.0 mg, 2.7 mmol), which were dissolved, in air, in 1.0 mL formic acid. The mixture was allowed to sit overnight. The light orange homogeneous solution was then heated to 90 °C for 1 h forming 840 mL of gas. Gratifyingly, the IR spectrum of the gaseous products shows much lower levels of CO. Comparing the CO signal from the gaseous products to an independently-prepared ca. 10 ppm CO solution in air, we estimate that the CO produced is less than 10 ppm.

Dehydrogenation Products at 70 °C from a Saturated Sodium Formate Solution
(Zoomed In)

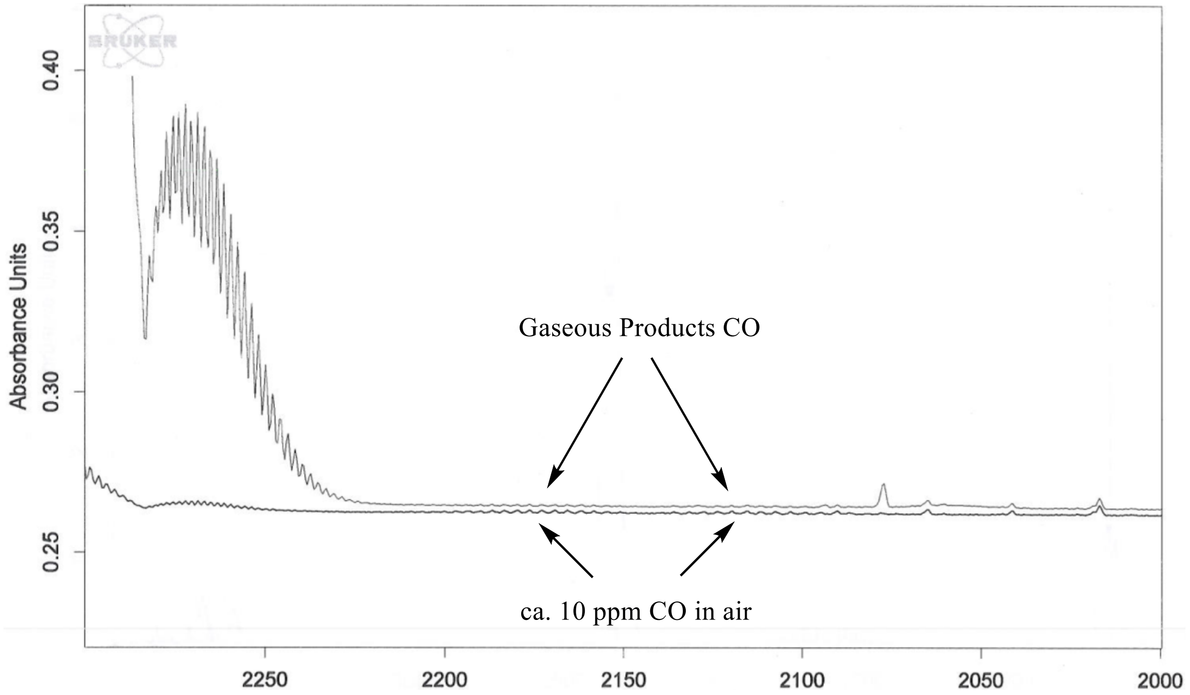

Supplementary Figure 6 | Comparison of IR spectra of gaseous products from supersaturated sodium formate solution of neat FA heated at 70 °C for 6 h and ca. 10 ppm CO in air matrix. We reasoned that we might be able to decrease the CO production using neat formic acid by using solutions that are highly concentrated in sodium formate. Thus, a solution of 30 mg (44 μmol) of the iridium precatalyst and 900 mg of the sodium formate in 1 mL formic acid was prepared in the drybox and allowed to sit overnight. The mixture was then taken out of the drybox and exposed to air. The supersaturated, very viscous mixture was heated to 70 °C without stirring for 6 h, producing 550 mL of gaseous products. IR analysis of the gaseous products shows levels of CO below 10 ppm.

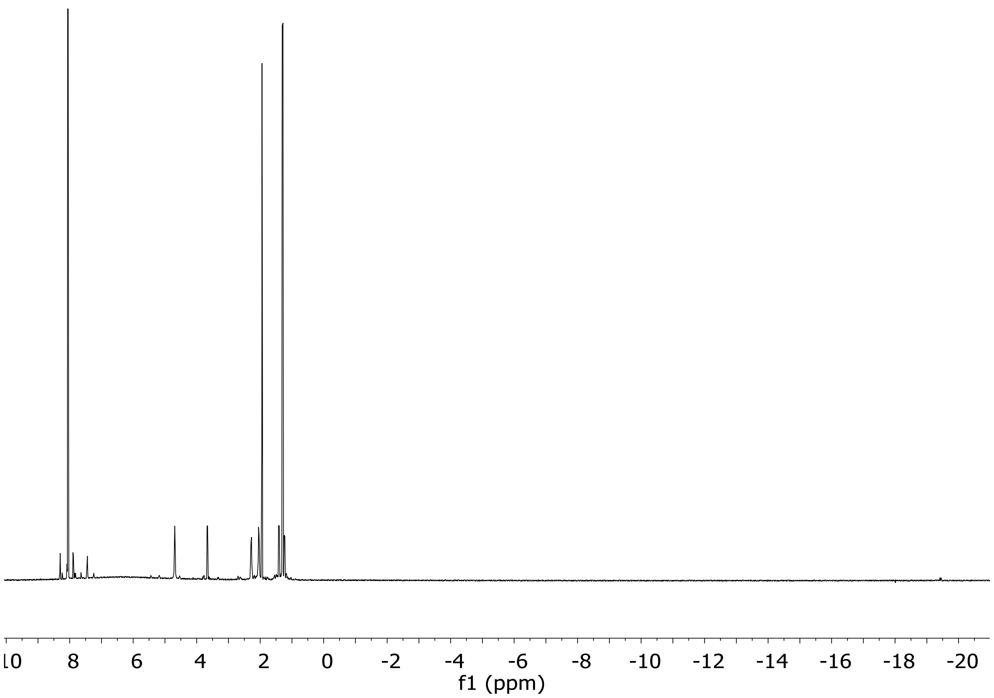

Supplementary Figure 7 | ^{1}H NMR spectrum of precatalyst 1 ca. 15 min after addition of 1 equiv. of sodium formate and 10 equiv formic acid (CD_3CN solvent). See Supplementary Discussion for analysis of the conversion of complex **1** to complex **2** in acetonitrile solvent.

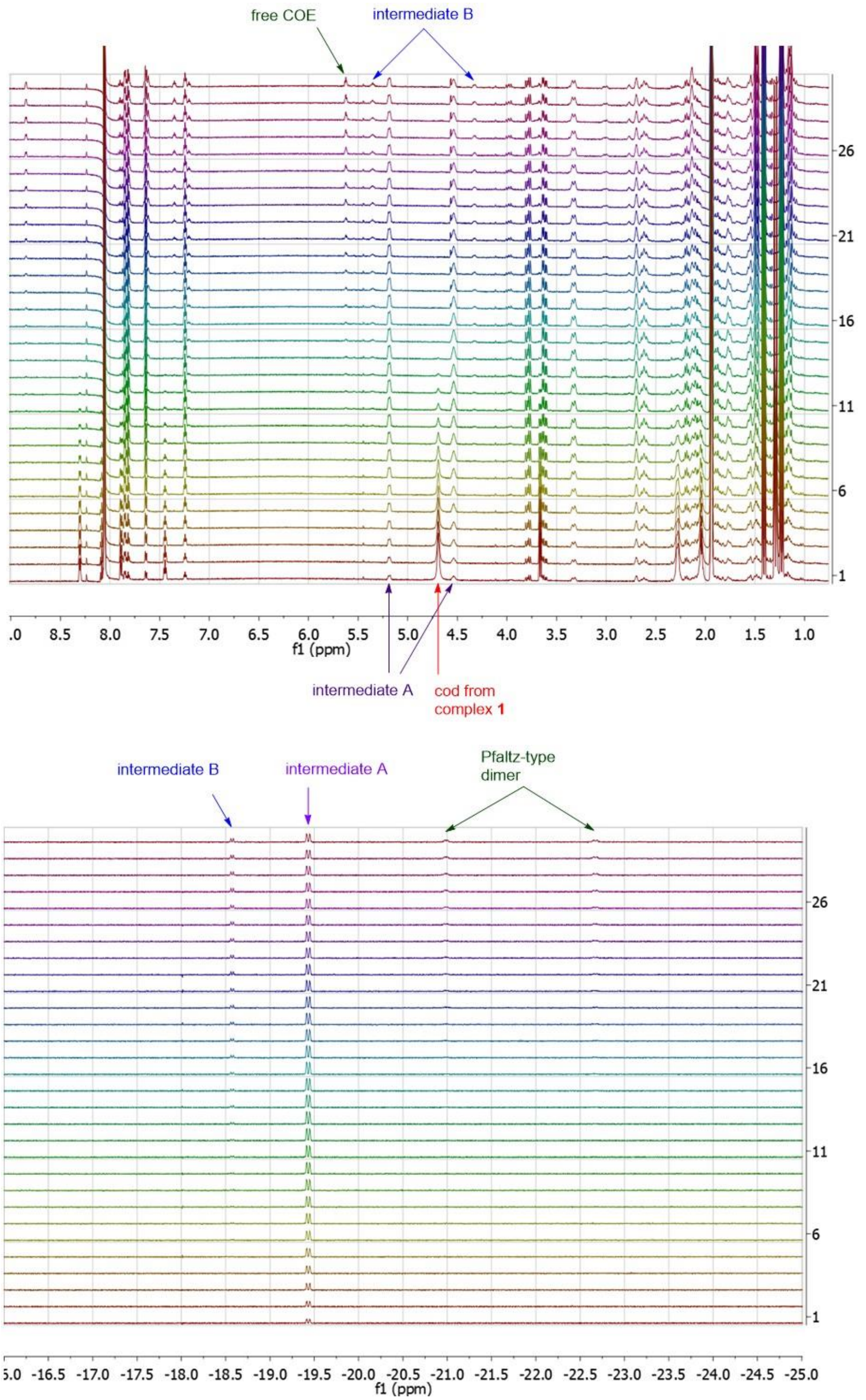

Supplementary Figure 8 | ^{1}H NMR time course experiment began ca. 20 min after addition of 1 equiv of sodium formate and 10 equiv formic acid (CD_3CN solvent). Top: aliphatic region. Bottom: hydride region. See Supplementary Discussion for analysis of the conversion of complex **1** to complex **2** in acetonitrile solvent.

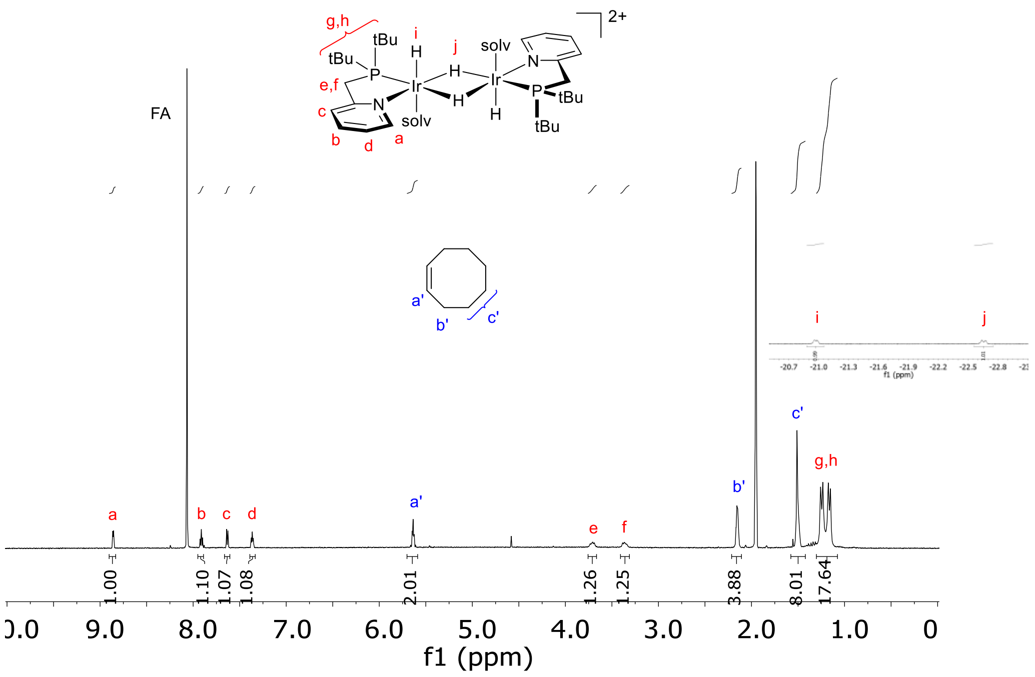

Supplementary Figure 9 | ¹H NMR spectrum of the products in the reaction of precatalyst 1 with FA in CD_3CN: a Pfaltz-type dimer and cyclooctene. See Supplementary Discussion for analysis of the conversion of complex **1** to complex **2** in acetonitrile solvent.

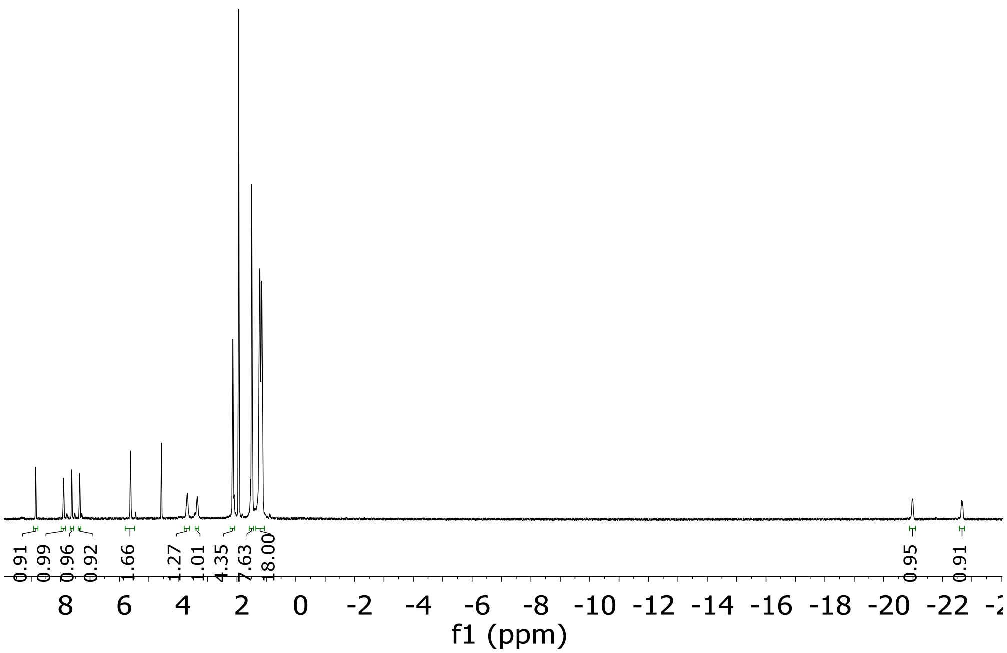

Supplementary Figure 10 | ¹H NMR spectrum of the products in the reaction of precatalyst 1 with H_2 in CD_3CN: a Pfaltz-type dimer and cyclooctene. See Supplementary Discussion for analysis of the conversion of complex **1** to complex **2** in acetonitrile solvent.

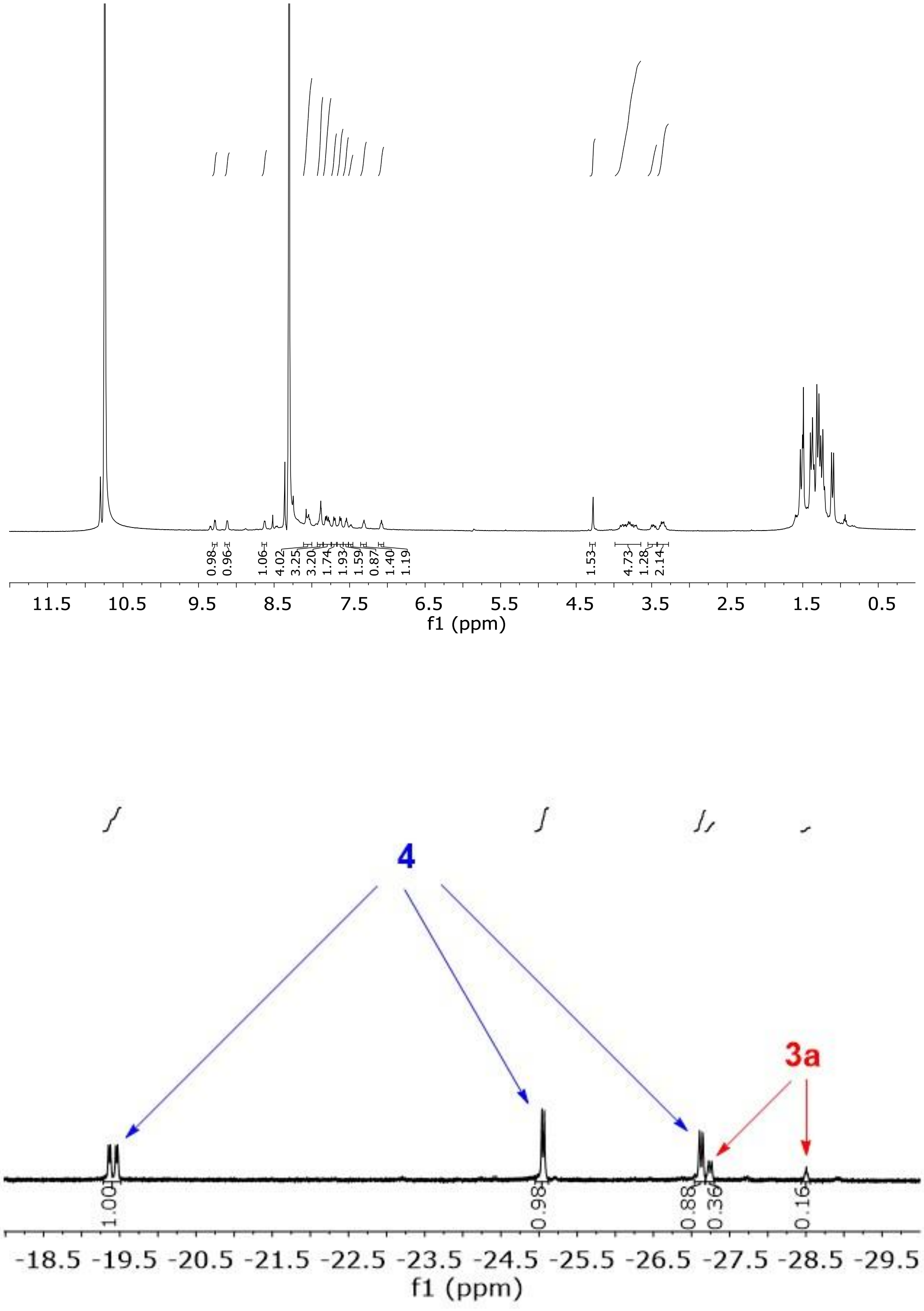

Supplementary Figure 11 | ^{1}H NMR spectrum of the iridium catalyst in formic acid. The bottom, hydride region, shows complexes 3a and 4. See Supplementary Discussion for analysis of the conversion of complex **1** to complexes **3a** and **4** in formic acid solvent.

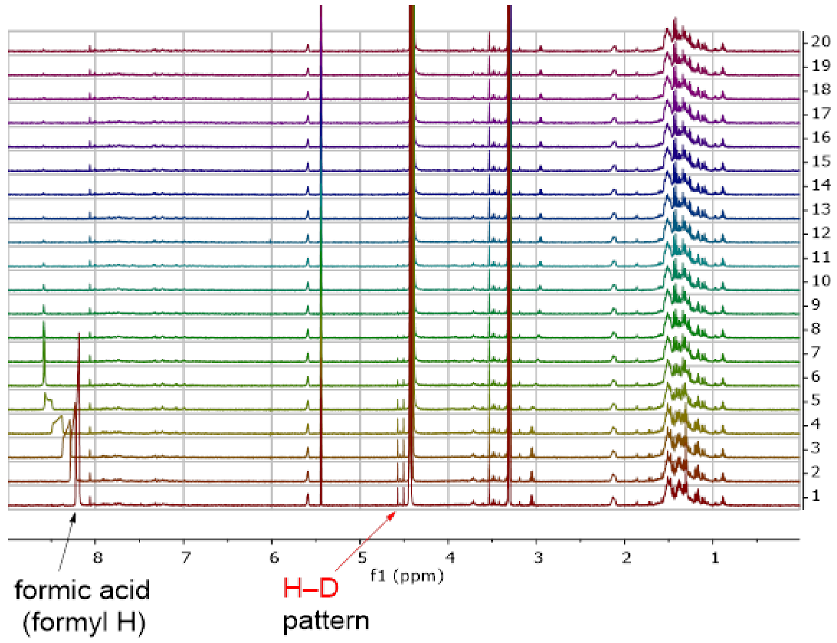

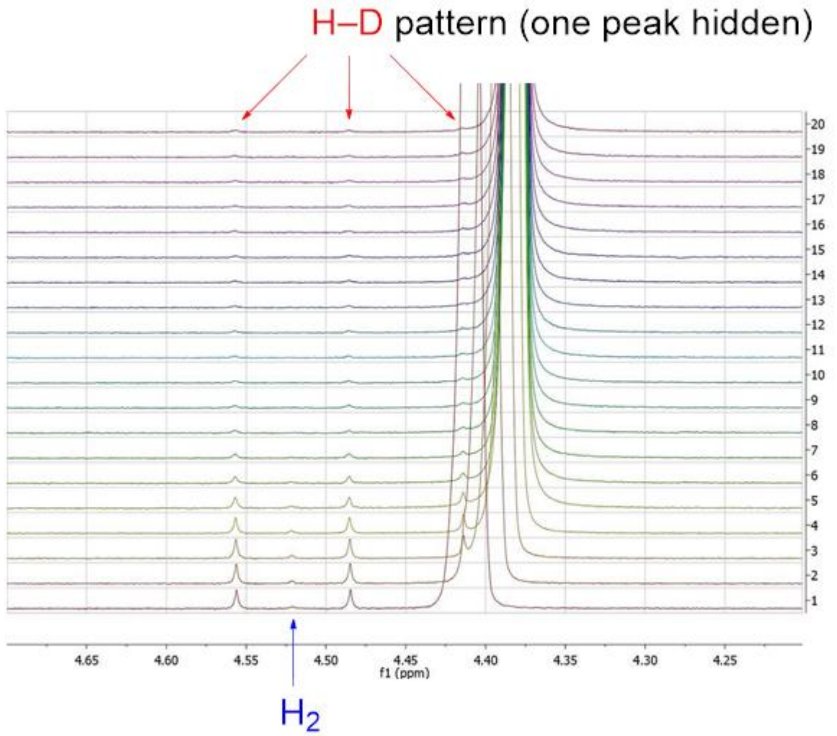

Supplementary Figure 12 | Time course ^{1}H NMR experiment showing H-D formation. The bottom, zoomed in region, more clearly shows H-D formation. See Supplementary Discussion for analysis of the proton-hydride fidelity experiment.

A.

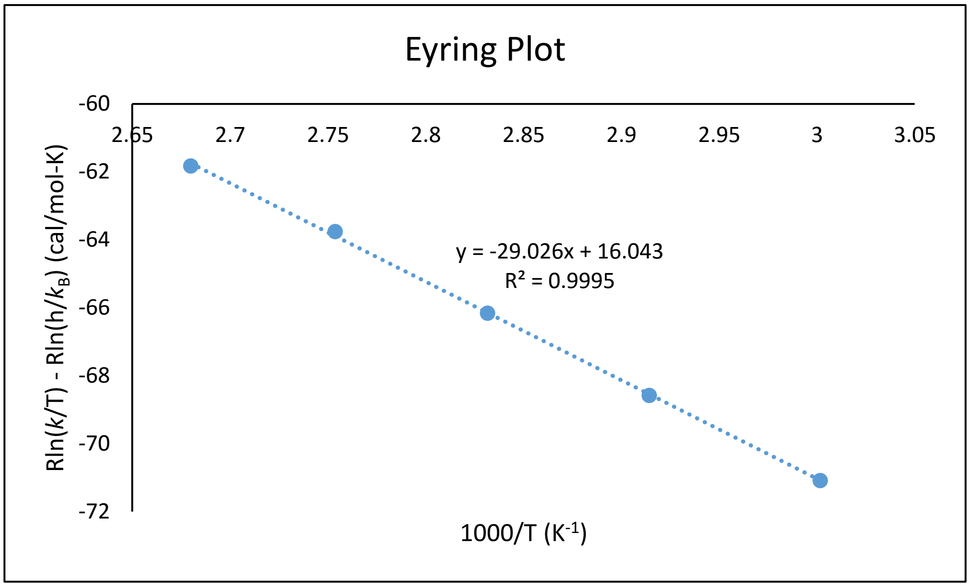

B.

Temperature (°C)	Rate (10^{-9} mol s^{-1})[a]	Turnover Frequency (10^{-3} s^{-1})[b]
60	2.5(2)	2.0
70	9(1)	7.2
80	32(2)	24.9
90	109(3)	86.1
100	296(5)	233.8

[a]Obtained at 86 °C; average of two runs, error is one standard deviation. [b]Obtained by dividing the rate of formic acid decomposition with the moles of iridium precatalyst in a 0.5 mL aliquot of the stock solution (2.52 mM in iridium).

Supplementary Figure 13 | A. Eyring plot for dehydrogenation. B. Data used to construct Eyring plot. In the drybox, a formic acid solution that is 5 mol% in sodium formate was prepared by dissolving sodium formate (901 mg, 13.2 mmol) in formic acid (10.0 mL, 265 mmol). To this solution was added the iridium precatalyst (18.2 mg, 26.2 μmol) to make a stock solution of the catalyst (2.52 mM based on monoiridium). The solution is allowed to sit overnight before use. The initial rates of formic acid decomposition were obtained using Procedure 2 (see Methods). An Eyring plot was constructed utilizing the measured turnover frequencies. From the Eyring plot, $\Delta H^{\ddagger}$ = +29.0(4) kcal mol^{-1} and $\Delta S^{\ddagger}$ = +16.0(10) eu.

A.

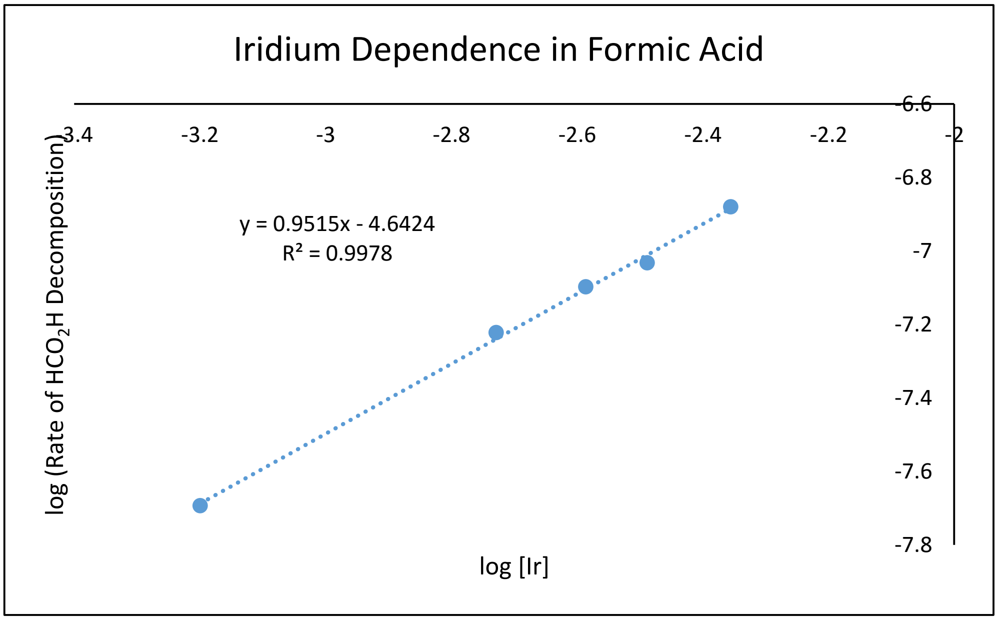

B.

Additional mass of Ir added (mg)	[Ir] in reaction mixture (mM)	[NaO_2CH] in reaction mixture (M)	[FA] in reaction mixture (M)	Rate of HCO_2H Decomposition (10^{-8} mol s^{-1}) [a]
0	0.63	0.63	25.9	2.0(1)
1.7	1.86	0.63	25.9	6.0(6)
2.7	2.59	0.63	25.9	8.0(2)
3.6	3.25	0.63	25.9	9.3(5)
5.2	4.41	0.63	25.9	13(1)

[a] Obtained at 86 °C; average of two runs, error is one standard deviation.

Supplementary Figure 14 | A. Plot of log (rate of formic acid decomposition) versus log [iridium] (formic acid solvent). B. Data used to construct log/log plot for iridium dependence in formic acid. In the drybox, a formic acid solution that is 5 mol% in sodium formate was prepared by dissolving sodium formate (901 mg, 13.2 mmol) in formic acid (10.0 mL, 265 mmol). To this solution was added the iridium precatalyst (9.1 mg, 13.1 µmol) to make a stock solution that is 0.005 mol% in the precatalyst (1.26 mM, 50 ppm, based on iridium atom). To set-up solutions for kinetics experiments, in the drybox, 1.0 mL of the catalyst stock solution is transferred via syringe to a 2 mL volumetric flask. A weighed amount of solid catalyst is added (figure B). Formic acid is then added to make 2.00 mL of solution. The initial rates of formic acid decomposition were obtained using Procedure 2 (see Methods). A plot of log (rate of formic acid decomposition) versus log [Ir] shows that the reaction order is 0.95(3) in iridium catalyst (figure A; the error is standard error of fit). Thus, the reaction is first order in iridium.

A.

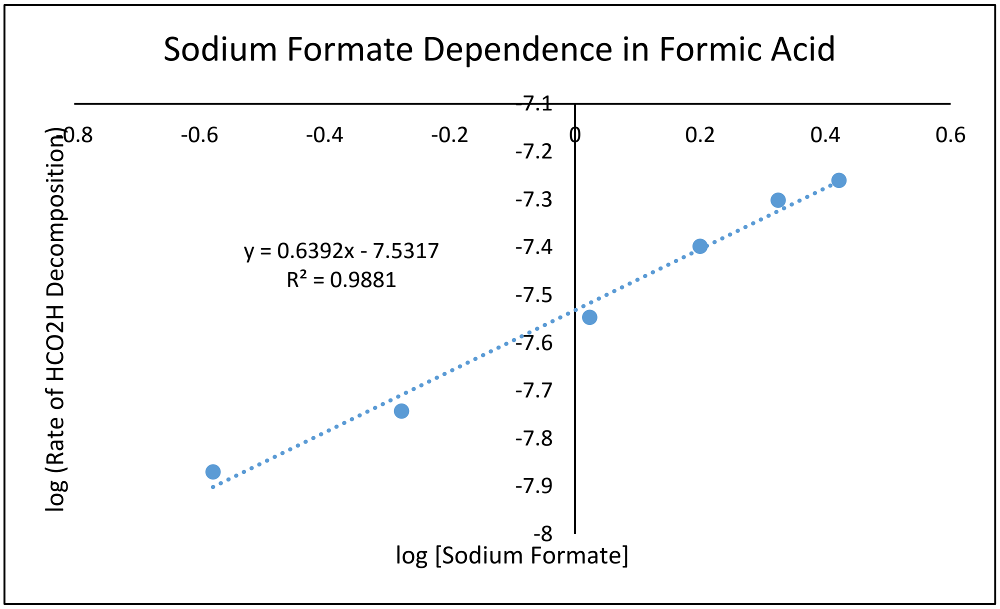

B.

Added NaO_2CH (mg)	[Ir] (mM)	[NaO_2CH] (M)	[FA] (M)	Rate (10^{-8} mol s^{-1}) [a]
0	0.66	0.26	26.26	1.4(3)
72	0.66	0.53	26.01	1.8(1)
144	0.66	1.06	25.51	2.8(1)
216	0.66	1.59	25.01	3.6(2)
288	0.66	2.11	24.51	5.3(1)
360	0.66	2.65	24.01	6.0(4)

[a] Obtained at 86 °C; average of two runs, error is one standard deviation.

Supplementary Figure 15 | A. Plot of log (rate of formic acid decomposition) versus log [sodium formate] (formic acid solvent). B. Data used to construct log/log plot for sodium formate dependence in formic acid. In the drybox, a formic acid stock solution that is 0.53 mM in sodium formate was prepared by dissolving sodium formate (360 mg, 5.3 mmol) in formic acid (10.0 mL, 265 mmol). To this solution was added iridium complex **1** (9.1 mg, 13.1 μmol). To set up solutions for kinetics experiments, 1.0 mL of the catalyst stock solution is transferred via syringe to a 2 mL volumetric flask in the drybox. A portion of sodium formate is added. Formic acid is then added to make 2.00 mL of solution. The initial rates of formic acid decomposition were obtained using Procedure 2 (see Methods). A plot of log (rate of formic acid decomposition) versus log [NaO_2CH] shows that the reaction order is 0.64(5) in sodium formate. Thus, the reaction is half-order in NaO_2CH.

A.

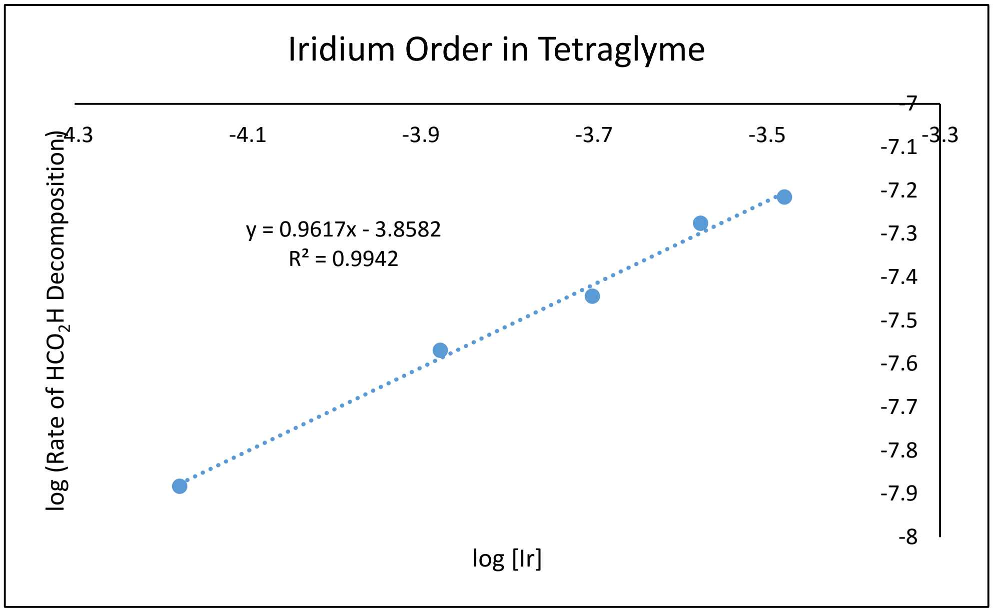

B.

FA added	$(n\text{Bu})_4\text{N(OH)}$ solution	Ir solution	[Ir] (μM)	$[(n\text{Bu})_4\text{N}(\text{O}_2\text{CH})]$ (mM)	[FA] (mM)	Rate (10^{-8} mol s^{-1}) [a]
21 μL, 0.53 mmol	22 μL, 26.4 μmol	50 μL, 0.13 μmol	66	13.2	265	1.3(1)
21 μL, 0.53 mmol	22 μL, 26.4 μmol	100 μL 0.26 μmol	130	13.2	265	2.7(2)
21 μL, 0.53 mmol	22 μL, 26.4 μmol	150 μL 0.39 μmol	200	13.2	265	3.6(2)
21 μL, 0.53 mmol	22 μL, 26.4 μmol	200 μL 0.52 μmol	260	13.2	265	5.3(1)
21 μL, 0.53 mmol	22 μL, 26.4 μmol	250 μL 0.65 μmol	330	13.2	265	6.1(1)

[a] Obtained at 86 °C; average of two runs, error is one standard deviation.

Supplementary Figure 16 | A. Plot of log (rate of formic acid decomposition) versus log [iridium] (tetraglyme solvent). B. Data used to construct log/log plot for iridium dependence in tetraglyme. In the drybox, a stock solution of iridium in tetraglyme (2.64 mM) was prepared by dissolving the iridium precatalyst **1** (18.2 mg, 26.46 mmol) in a volumetric flask to make 10.00 mL of stock solution in tetraglyme. Because sodium formate is insoluble in tetraglyme, we generated soluble $(n\text{-Bu})_4\text{N}(\text{HCO}_2)$ by deprotonating formic acid with 1.20 M $(n\text{-Bu})_4\text{N(OH)}$ in methanol. $(n\text{-Bu})_4\text{N(OH)}$ was titrated using benzoic acid and bromothymol blue as indicator. To set-up solutions for kinetics experiments, a 2 mL volumetric flask is filled with ca. 1 mL of tetraglyme in the drybox. Then 22 μL of 1.21 M $(n\text{-Bu})_4\text{N(OH)}$ in methanol and 21 μL of formic acid are added and the solution thoroughly mixed. A measured amount of the iridium catalyst stock solution (figure B) is added and the solution thoroughly mixed. The color of the solution changes immediately from orange to very pale yellow. Tetraglyme is then added to make 2.00 mL of solution. Using this freshly prepared solution, the initial rates of formic acid decomposition were obtained using Procedure 2 (see Methods), except that a Schlenk test tube with a side-arm is utilized instead of a 5 mL high pressure reaction vessel. A plot of log (rate of formic acid decomposition) versus log [Ir] shows that the reaction order is 0.96(4) in iridium catalyst. Thus, the reaction is first order in iridium.

A.

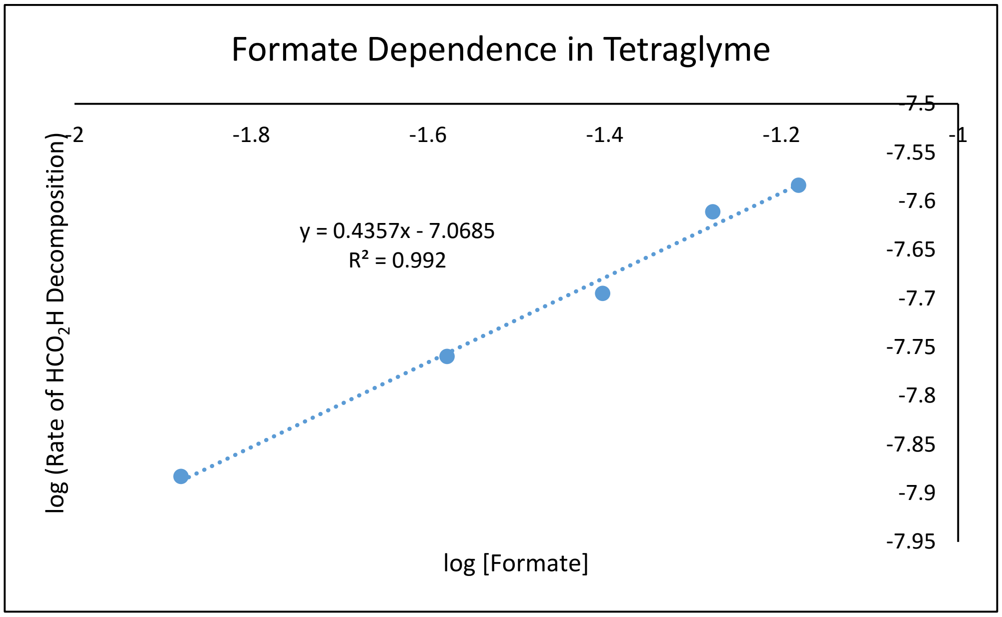

B.

FA added	(nBu)$_4$N(OH) solution	Ir solution	[Ir] (µM)	[(nBu)$_4$N(O_2CH)] (mM)	[FA] (mM)	Rate (10^{-8} mol s^{-1}) [a]
21 µL, 0.53 mmol	22 µL, 26.4 µmol	100 µL 0.13 µmol	66	13.2	265	1.3(1)
22 µL, 0.53 mmol	44 µL, 52.8 µmol	100 µL 0.13 µmol	66	26.4	265	1.7(1)
23 µL, 0.53 mmol	66 µL, 79.2 µmol	100 µL 0.13 µmol	66	39.6	265	2.0(1)
24 µL, 0.53 mmol	88 µL, 105.6 µmol	100 µL 0.13 µmol	66	52.8	265	2.4(1)
25 µL, 0.53 mmol	110 µL, 132 µmol	100 µL 0.13 µmol	66	66.0	265	2.6(1)

[a] Obtained at 86 °C; average of two runs, error is one standard deviation.

Supplementary Figure 17 | A. Plot of log (rate of formic acid decomposition) versus log [formate] (tetraglyme solvent). B. Data used to construct log/log plot for (n-Bu)$_4$N(O_2CH) in tetraglyme. In the drybox, a stock solution of the iridium precatalyst in tetraglyme (1.32 mM) was prepared by dissolving the iridium precatalyst (9.1 mg, 13.23 mmol) in a volumetric flask to make 10.00 mL of solution. Using this stock solution, kinetics data were obtained according to the procedure described in Supplementary Figure 16 except that (n-Bu)$_4$N(O_2CH) concentration was varied instead of iridium concentration. Also, methanol was added so that each solution had the same amount of methanol. A log/log plot of rate of formic acid decomposition versus (n-Bu)$_4$N(O_2CH) concentration shows a reaction order of 0.44(2). This indicates a reaction order of 0.5 with respect to (n-Bu)$_4$N(O_2CH).

A.

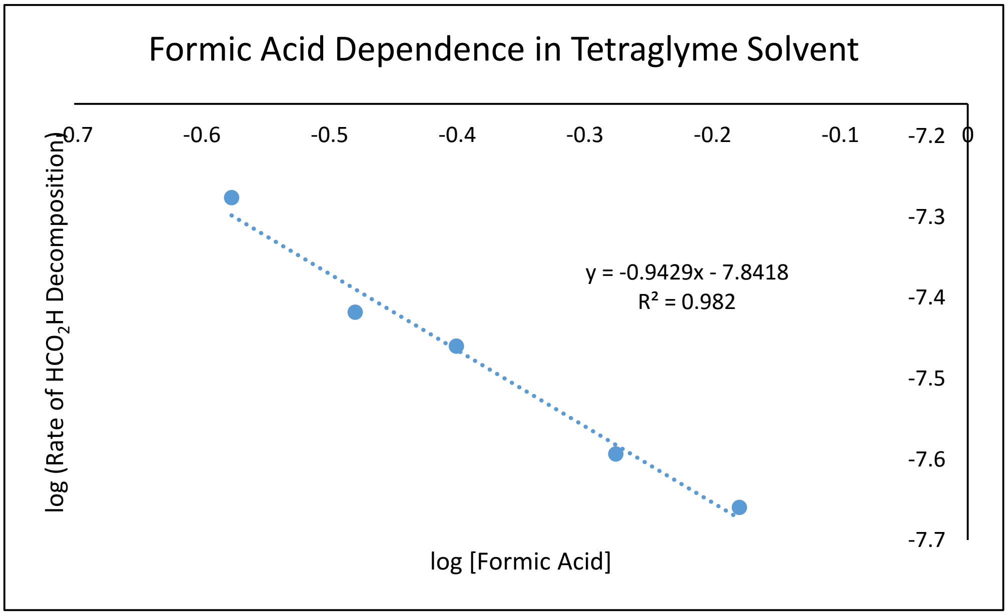

B.

FA added	(nBu)$_4$N(OH) solution	Ir solution	[Ir] (μM)	[(nBu)$_4$N(O$_2$CH)] (mM)	[FA] (mM)	Rate (10^{-8} mol s^{-1})[a]
21 μL, 0.53 mmol	22 μL, 26.4 μmol	200 μL 0.52 μmol	260	13.2	265	5.3(1)
26 μL, 0.66 mmol	22 μL, 26.4 μmol	200 μL 0.52 μmol	260	13.2	331	3.8(2)
31 μL, 0.80 mmol	22 μL, 26.4 μmol	200 μL 0.52 μmol	260	13.2	398	3.5(1)
41 μL, 1.06 mmol	22 μL, 26.4 μmol	200 μL 0.52 μmol	260	13.2	530	2.6(1)
51 μL, 1.32 mmol	22 μL, 26.4 μmol	200 μL 0.52 μmol	260	13.2	662	2.2(1)

[a] Obtained at 86 °C; average of two runs, error is one standard deviation.

Supplementary Figure 18 | A. Plot of log (rate of formic acid decomposition) versus log [formic acid] (tetraglyme solvent). B. Data used to construct log/log plot for formic acid dependence in tetraglyme. In the drybox, a stock solution of the iridium precatalyst in tetraglyme (2.64 mM) was prepared by dissolving the iridium precatalyst (18.2 mg, 26.46 mmol) in a volumetric flask to make 10.00 mL of solution. Using this stock solution, kinetics data were obtained according to the procedure described in Supplementary Figure 16 except that formic acid concentration was varied instead of iridium concentration. A log/log plot of rate of formic acid decomposition versus formic acid concentration shows a reaction order of -0.94(9). This indicates a reaction order of -1.0 with respect to formic acid.

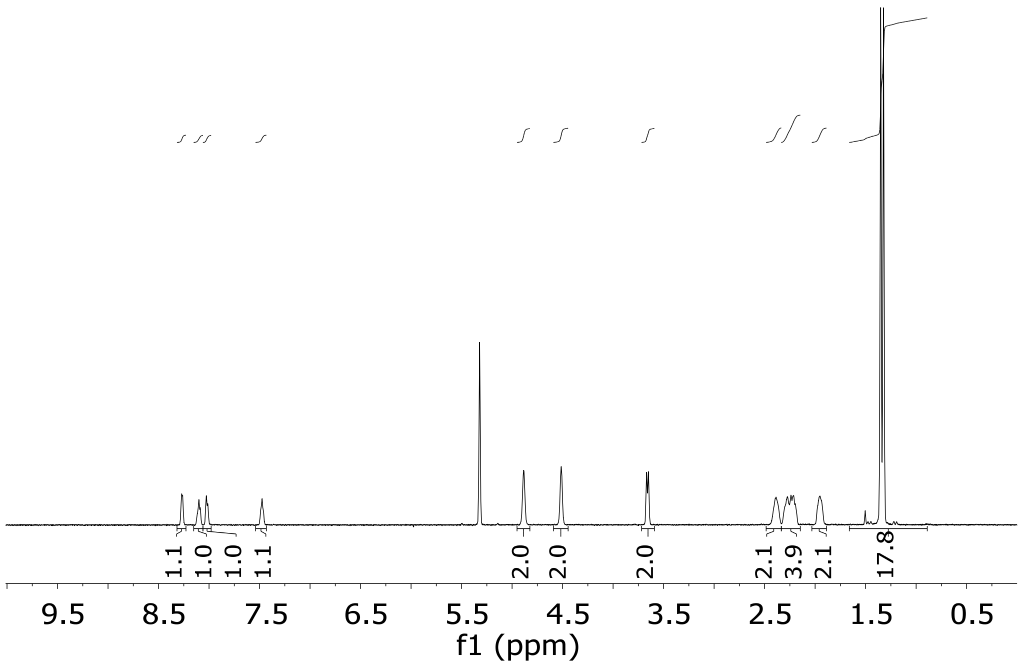

Supplementary Figure 19 | ^{1}H NMR spectrum of complex 1 at 25 °C in CD_2Cl_2.

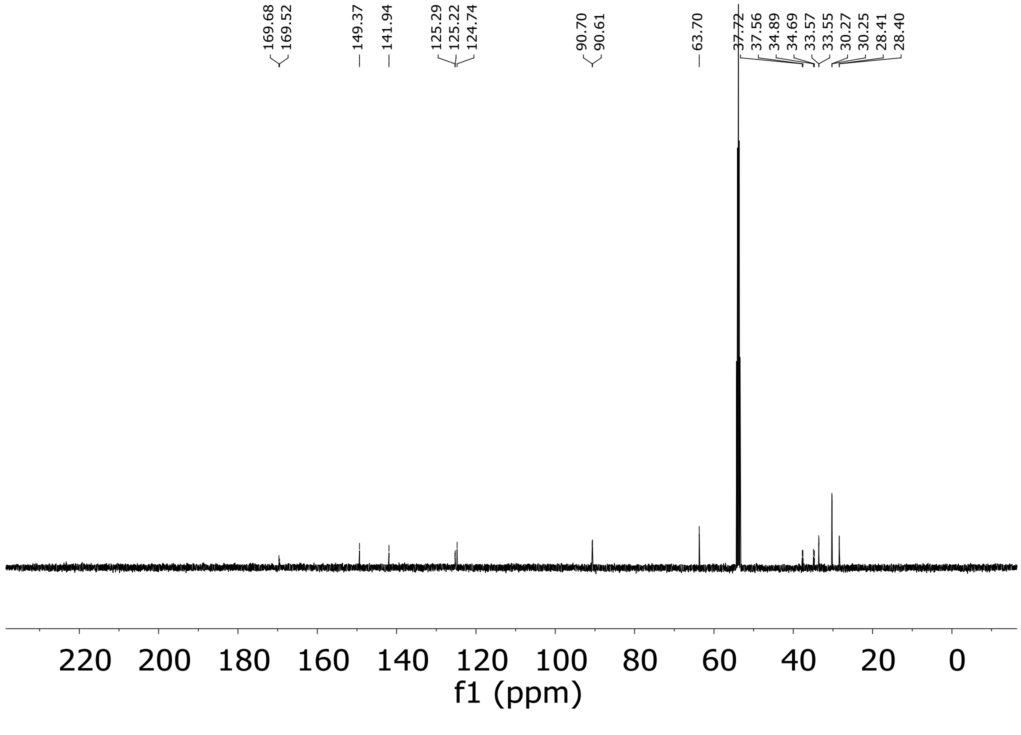

Supplementary Figure 20 | ^{13}C NMR spectrum of complex 1 at 25 °C in CD_2Cl_2.

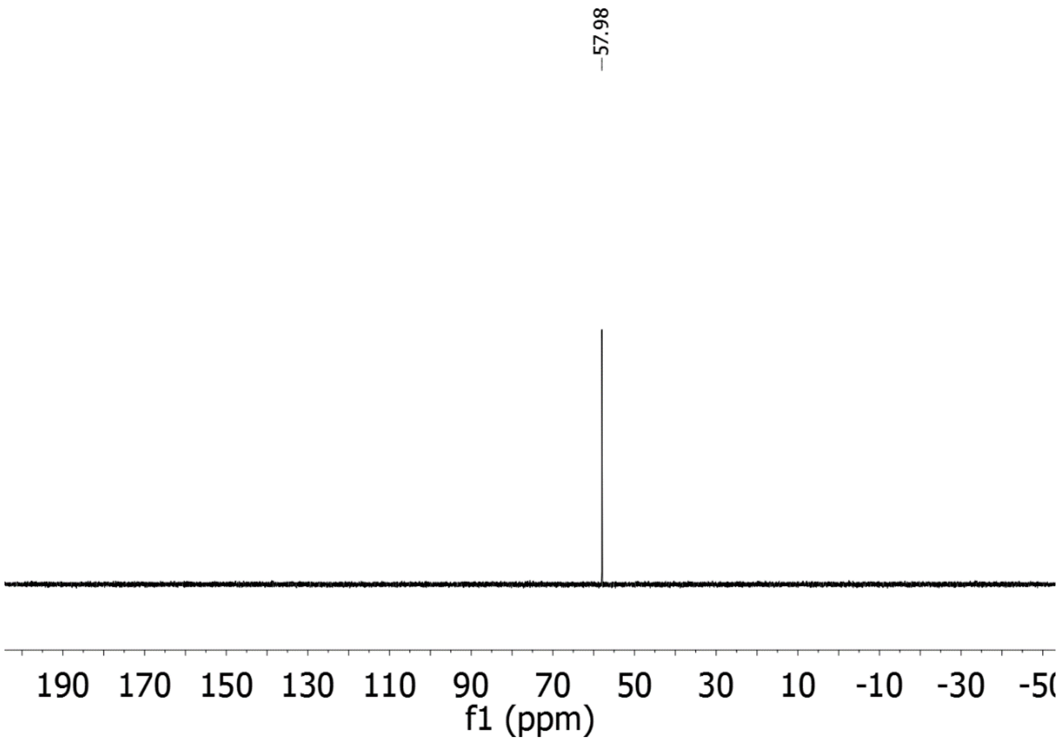

Supplementary Figure 21 | ^{31}P NMR spectrum of complex 1 at 25 °C in CD_2Cl_2.

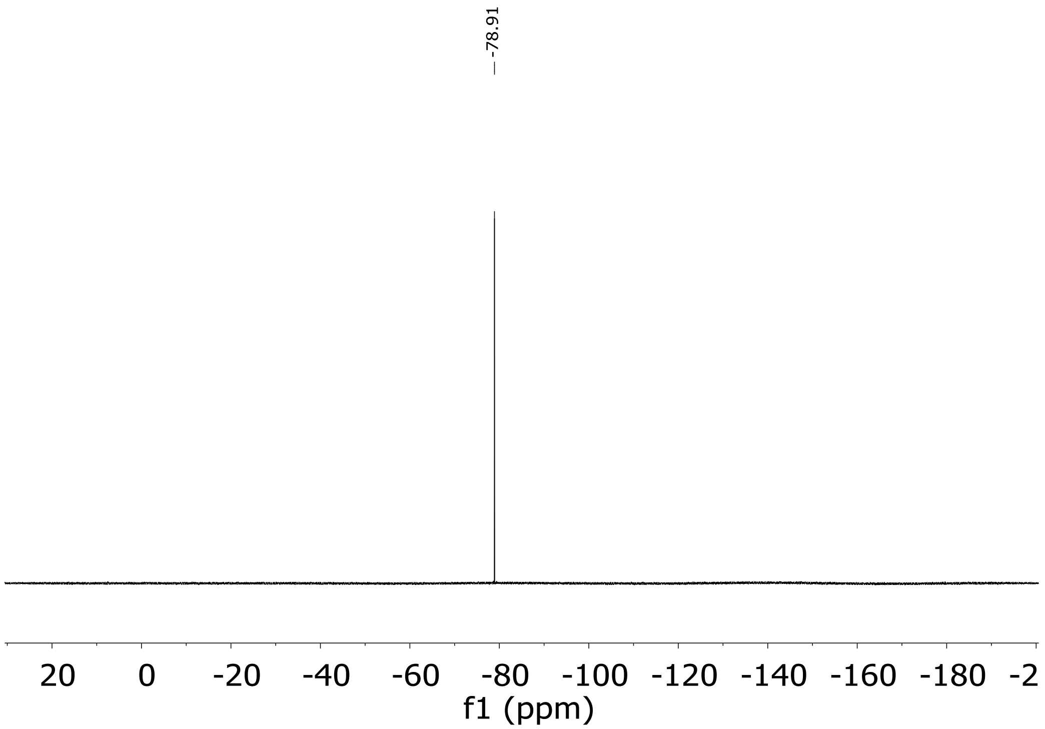

Supplementary Figure 22 | ^{19}F NMR spectrum of complex 1 at 25 °C in CD_2Cl_2.

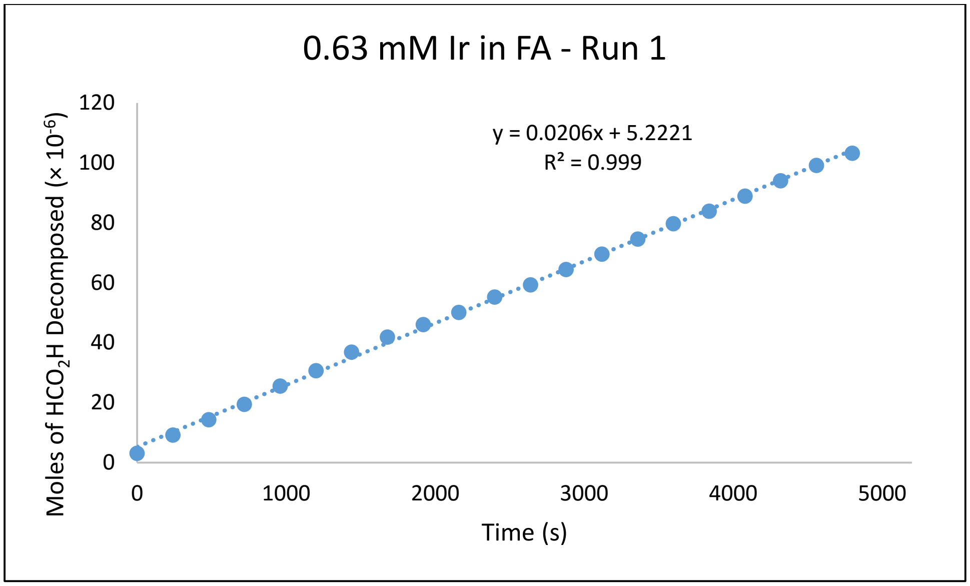

Supplementary Figure 23 | Sample plot of moles of formic acid decomposed versus time (formic acid solvent).

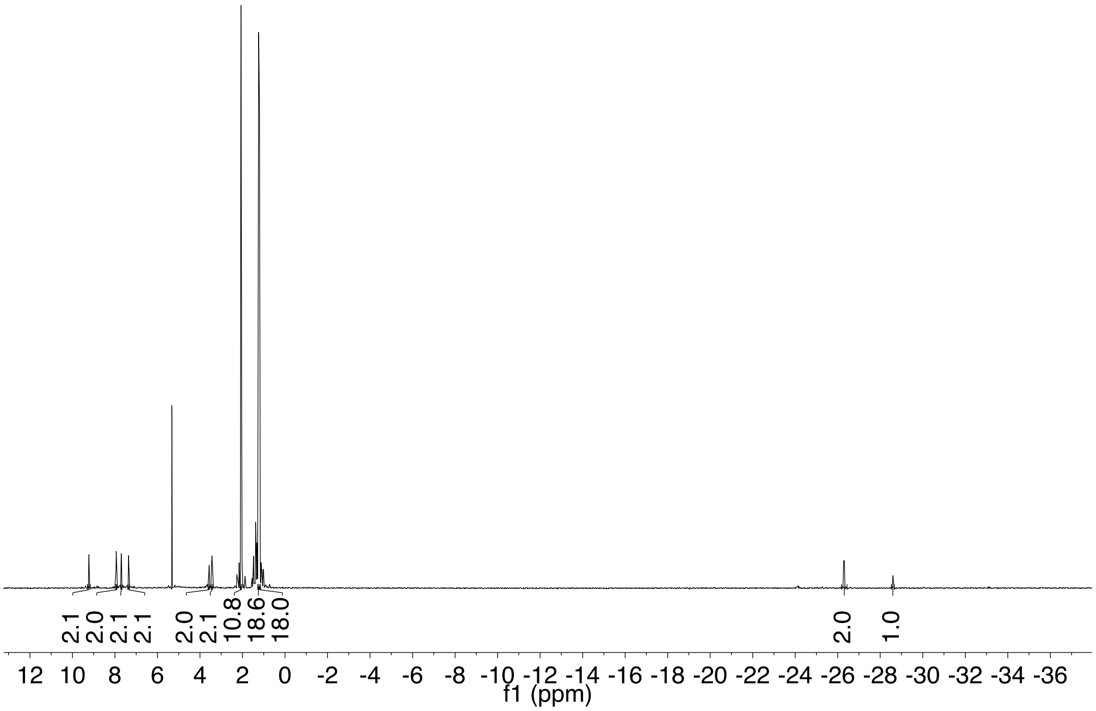

Supplementary Figure 24 | ^{1}H NMR spectrum of complex 3b at 25 °C in CD_2Cl_2.

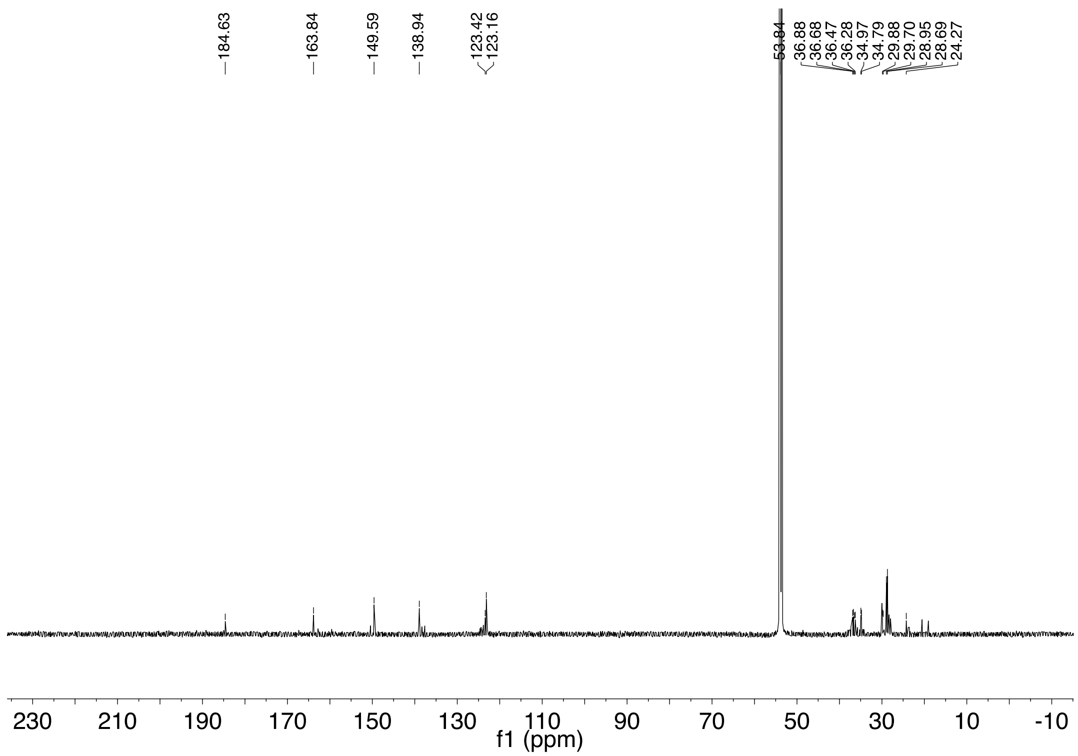

Supplementary Figure 25 | ^{13}C NMR spectrum of complex 3b at 25 °C in CD_2Cl_2.

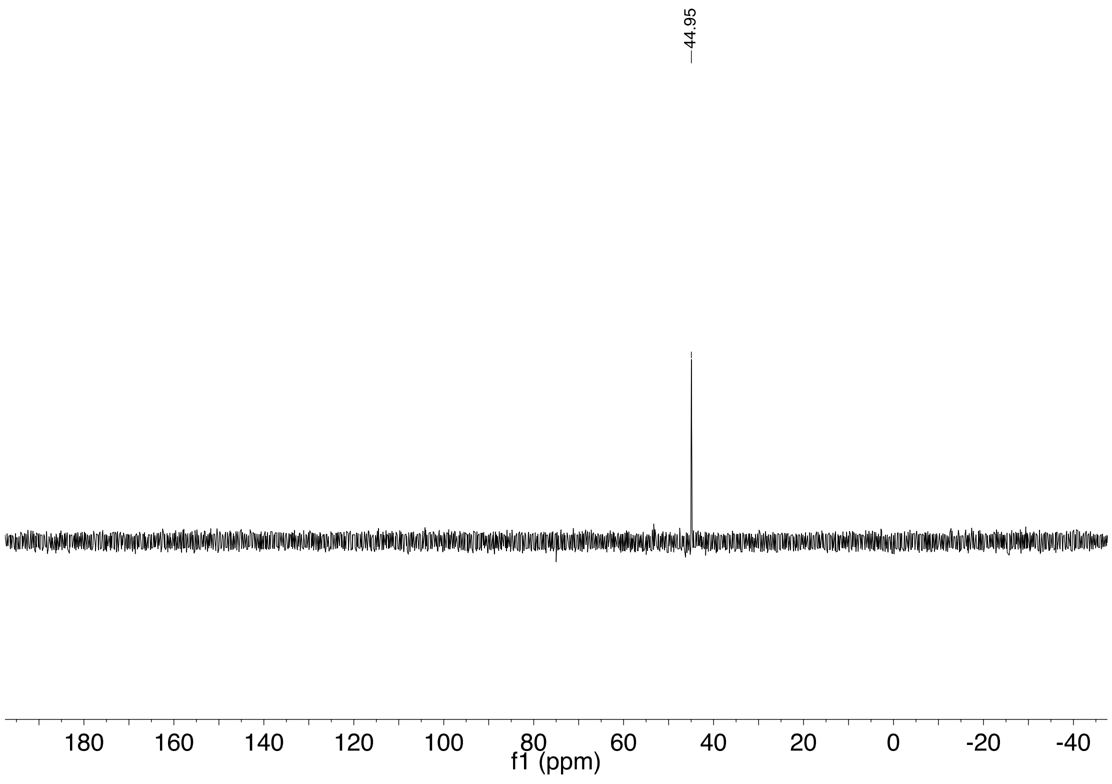

Supplementary Figure 26 | ^{31}P NMR spectrum of complex 3b at 25 °C in CD_2Cl_2.

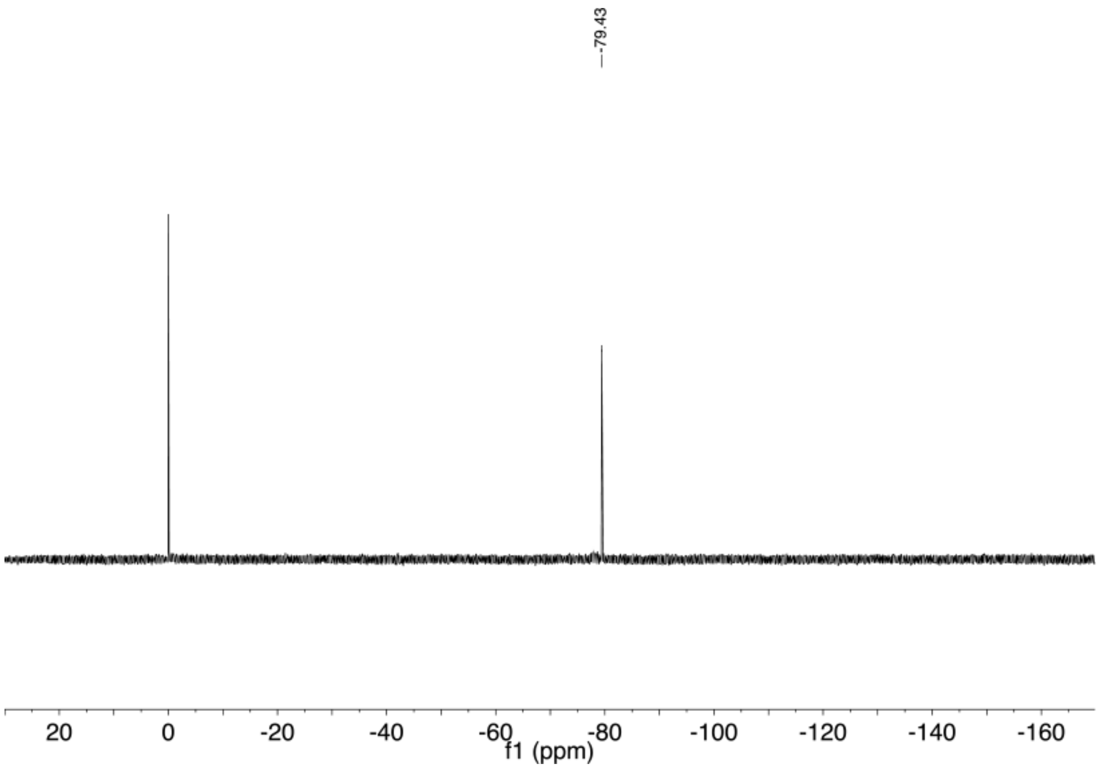

Supplementary Figure 27 | ^{19}F NMR spectrum of complex 3b at 25 °C in CD_2Cl_2.

Supplementary Tables

Supplementary Table 1 | Selected Homogeneous Catalysts for Formic Acid Dehydrogenation.

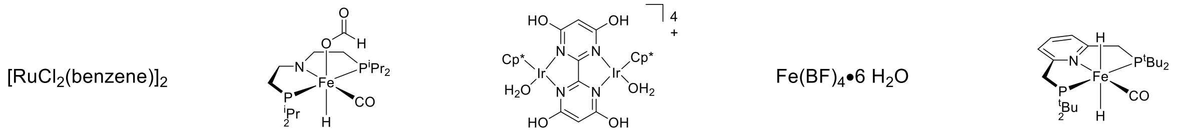

I Beller | II Bernskoetter, Hazari, & Schneider | III Hull & Fujita | IV Beller | V Milstein

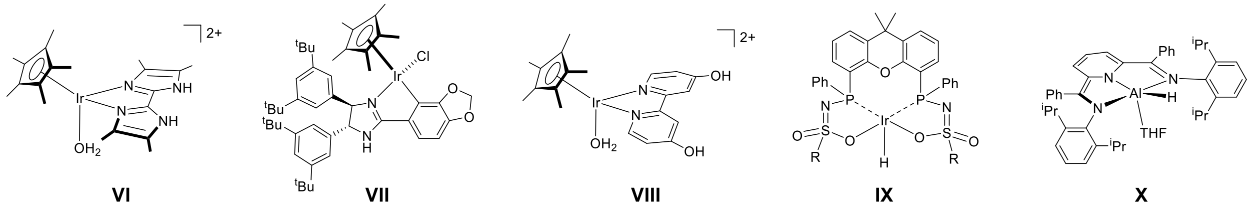

VI Himeda | VII Xiao | VIII Himeda | IX Reed | X Berben

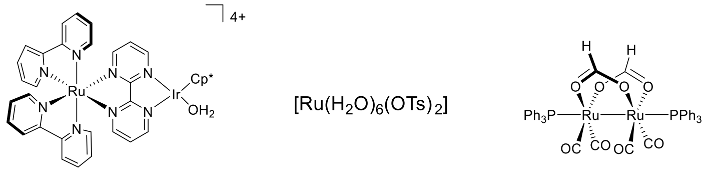

XI Fukuzumi | XII Dyson & Laurenczy | XIII Wills

Catalyst	Solvent / Medium	Additive	T (°C)	TON	max TOF (h^{-1})	CO produced	Ref.
**1**	Neat FA	SF	90	2.16 M	13,000	(vide infra)	This work
**I**	FA/Et_3N	dppe	25	>1 M	1000	< 2 ppm	6
**II**	dioxane	10% $LiBF_4$	80	983,642	196,728	9 ppm	1
**III**	1 M 1:1 FA/SF	SF	90	308,000	228,000	ND	7
**IV**	PC	3 equiv PP_3	80	92,417	9,425	ND	2
**IV**	PC	*rac*-P_4	60	6061	1737	ND	8
**V**	THF	Et_3N	40	100,000	836	ND	9
**VI**	1 M aq. FA	None	80	10,000	34,000	ND	10
**VII**	5:2 FA/Et_3N	Et_3N	40	NR	147,000	ND	11
**VIII**	2 M aq. FA	None	90	NR	14,000	ND	12
**IX**	Dioxane	None	85	3000	3271	ND	13
**X**	THF	Et_3N	65	2200	5200	ND	14
**XI**	H_2O	SF	25	142	426	ND	15
**XII**	H_2O	SF, TPPTS	120	NR	460	ND	16
**XIII**	FA/ Et_3N	Et_3N	120	18,000	17,800	250 ppm	17

FA = formic acid; SF = sodium formate; PC = propylene carbonate; dppe = diphenylphosphinoethane; PP_3 = tris[2-diphenylphosphino)ethyl]phosphine; rac-P_4 = 1,1,4,7,10,10-hexaphenyl-1,4,7,10-tetraphosphadecane; TPPTS = 3,3',3''-phosphanetriyltris trisodium salt; ND = not detected; NR = not reported.

Supplementary Table 2 | Evaluation of the effect of different bases and different Lewis acids on formic acid dehydrogenation. Formic acid stock solutions that were 0.005 mol% (50 ppm) in [Ir atom] and 5 mol% in base were prepared in the drybox by adding measured amounts of the iridium catalyst (1.8 mg, 2.6 μmol) and base (see below for exact amounts) into a vial. Formic acid (2.0 mL) was then added to dissolve the base and the catalyst. These experiments were aimed to determine the effect of different bases on the rate of dehydrogenation. In addition, these experiments allowed evaluation of the effect on dehydrogenation by the Lewis acids Li^+, Na^+, K^+, and Ca^{2+}. The initial rates of formic acid decomposition (average of two runs) were obtained using Procedure 2 (see Methods). According to the table, base is required for dehydrogenation to occur efficiently. However, the types of bases and Lewis acids do not have an effect on the rate of dehydrogenation. One outlier, the data for calcium carbonate, gives a much lower decomposition rate because copious amounts of calcium formate precipitates.

**Base Used**	**Added Base (mg)**	**1 (ppm)**	**Base (mol%)**	**Rate ($\times 10^{-8}$ mol s^{-1})** [a]
None	0	50	0%	0.1 [b]
LiOH [c]	63	50	5%	4.7(1)
NaOH [c]	106	50	5%	5.2(1)
KOH [c]	149	50	5%	5.4(1)
NaO_2CH	180	50	5%	6.1(2)
KO_2CH	223	50	5%	6.4(2)
Na_2CO_3 [c]	140	50	2.5%	5.6(3)
K_2CO_3 [c]	183	50	2.5%	5.3(5)
$CaCO_3$ [c,d]	133	50	2.5%	2.5(1)

[a] Obtained at 86 °C; average of two runs, error is one standard deviation. [b] Average rate over 5 h; the rate decreases upon further heating. [c] 2.1 mL of formic acid was added; 0.1 mL reacts with the base. [d] Copious amounts of precipitates formed.

Supplementary Table 3 | Initial rates of dehydrogenation in the presence and absence of air. A formic acid stock solution that is 0.005 mol% in the iridium precatalyst and 5 mol% in sodium was prepared in air by adding 1.8 mg (2.6 μmol) of the iridium precatalyst and 180 mg (2.64 mmol) of sodium formate into a vial. In air, formic acid (2.0 mL) was then added to dissolve the base and the precatalyst. Dehydrogenation rates were obtained 1 day and 2 weeks after preparation of the stock solution using Procedure 2 (see Methods). Under otherwise identical conditions, the dehydrogenation of reaction mixtures prepared in air is ca. 3 times slower.

**Experiment**	**Iridium**	**NaO_2CH**	**Initial Rate (10^{-8} mol s^{-1})** [a]
Air [b]	0.005%	5%	2.1(5)
Air [c]	0.005%	5%	1.8(1)
No Air [b]	0.005%	5%	6.1(2)

[a] Obtained at 86 °C; average of two runs, error is one standard deviation. [b] Obtained 1 day after solution preparation. [c] Obtained 2 weeks after solution preparation.

Supplementary Table 4 | Catalyst reusability in air. A high pressure reaction flask possessing a side arm and a large bore plug valve was charged with the iridium catalyst (6.1 mg, 8.8 µmol) and sodium formate (184.1 mg, 2.7 mmol). In air, formic acid (0.5 mL, 13.2 mmol) was added. The color of the solution slowly changes from orange to pale yellow over the course of 1 hour. The flask was connected to a gas burette and heated to 90 °C. Formic acid decomposition was allowed to proceed until mostly solid catalysts remained. The volume of gas produced over time was recorded. The flask was then allowed to cool to room temperature, opened in air, and recharged with 0.5 mL of formic acid. This procedure was repeated dozens of times without significant loss in catalyst activity. After 50 loadings, 28.85 L of gas were produced, corresponding to a turnover number of 66,403 and 89% conversion.

Run	Volume (mL) [a]	Time (min) [b]	Run	Volume (mL) [a]	Time (min) [b]
1[c]	417	120	26	570	90
2	505	90	27	600	90
3[c]	512	150	28	610	90
4	513	120	29	580	75
5	560	120	30[a]	565	60
6	570	75	31	510	120
7	541	90	32	540	90
8	620	90	33	620	90
9	585	120	34	570	120
10[c]	619	90	35	610	120
11	607	80	36	600	75
12	550	60	37	600	75
13	605	120	38	590	120
14	600	90	39	565	90
15	605	130	40[a]	580	75
16	585	90	41	600	120
17	584	90	42	570	120
18	583	90	43	570	120
19	580	90	44	560	120
20[c]	595	80	45	590	120
21	549	150	46	580	120
22	540	110	47	640	120
23	580	90	48	650	120
24	580	80	49	600	120
25	530	90	50[a]	670	120

[a] Measured using a 1000 mL gas buret. [b] Heating was stopped when mostly solid sodium formate and catalyst was present in the reaction flask. [c]The initial rates and maximum turnover frequencies were measured (Table 1).

Supplementary Table 5 | High turnover number experiments. In the drybox, a 5 mL high pressure reaction flask with a side arm and a large bore plug valve was charged with sodium formate (0.18 g, 2.64 mmol), which was then dissolved in 0.6 mL of formic acid. A stock solution of the iridium precatalyst (which is actually a stock solution of the iridium dimer catalyst) in formic acid was added (100 μL, 1.26 mM precatalyst stock solution, 0.13 μmol precatalyst). The reaction flask was taken out of the drybox and connected to a vent line leading to a 1 L gas burette filled with oil. The flask was heated to 90 ^{o}C for 24 h. After 24 h, 420 mL of gas was produced, corresponding to 8.6 mmol of formic acid decomposed and a turnover number of 67,615. At the end of the reaction, a white solid residue remains at the bottom of the flask. Not all of the formic acid is decomposed because some of the formic acid ends up on the neck and the side arm of the flask and does not mix with the catalyst. Nevertheless, we can recharge the reaction flask with formic acid. To do this, we disconnect the sealed reaction flask from the gas burette, clean the side arm with acetone, and take the flask back into the drybox to be refilled with formic acid. Then we repeat this procedure through 40 cycles. After 40 cycles over a period of 4 months, 13.71 L of gas were produced. This corresponds to 0.28 mol of formic acid decomposed and 2.16 M turnovers. The catalyst has slowly lost its catalytic activity. After 4 months, ca. 15% of the initial catalytic activity remains.

**Run**	**Volume FA Loaded (mL)**	**Volume of Gas Produced (mL)** [a]	**Time (h)** [b]	**Run**	**Volume FA Loaded (mL)**	**Volume of Gas Produced (mL)** [a]	**Time (h)** [b]
1	0.6	420	24	21	0.2	160	20
2	0.6	490	21	22	0.2	110	16
3	0.8	560	32	23	0.2	140	18
4	0.6	500	26	24	0.6	360	72
5	1.0	800	60	25	0.6	360	72
6	0.6	420	27	26	0.6	300	72
7	0.5	450	27	27	0.6	310	72
8	0.5	310	22	28	0.6	320	72
9	0.5	410	36	29	0.6	360	80
10	0.8	480	60	30	0.6	290	72
11	0.3	290	30	31	0.6	380	80
12	0.3	290	24	32	0.6	320	72
13	0.2	230	16	33	0.6	400	80
14	0.2	210	20	34	0.6	330	72
15	0.8	460	70	35	0.6	380	96
16	0.3	200	24	36	0.6	300	72
17	0.3	220	90	37	0.6	400	120
18	0.2	160	18	38	0.6	360	120
19	0.2	180	20	39	0.6	310	120
20	0.6	440	72	40	0.6	300	120

[a] Measured using a 1000 mL gas burette. [b] Heating was stopped when mostly solid sodium formate and catalyst was present in the reaction flask.

Supplementary Discussions

Observation of catalytic intermediates by NMR.

Data regarding the elementary steps of the conversion of the precatalyst to the active dimer catalyst were obtained using NMR. We can observe the formation of a Pfaltz-type dimer **2** from precatalyst **1** in two different ways: 1) reaction of **1** with formic acid in a coordinating solvent such as acetonitrile or 2) reaction of **1** with H_2 in various solvents. In formic acid solvent, iridium dimer **2** is converted further into the (di-μ-formate)iridium dimer **3a**.

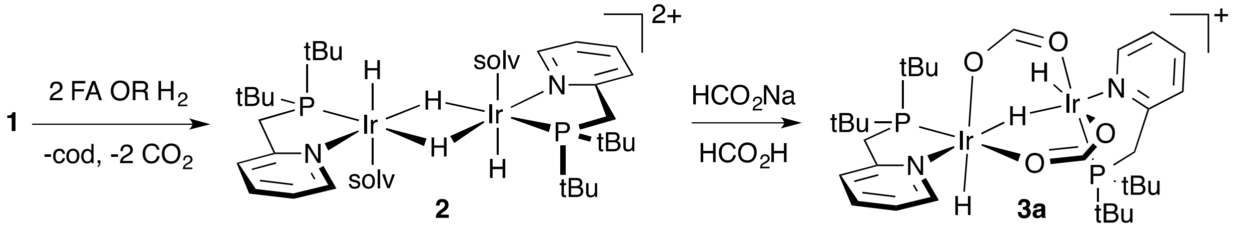

*Observation of Intermediate **2** in CD_3CN*

Room temperature ^{1}H NMR studies in CD_3CN show that addition of 1 equivalent of sodium formate and 10 equivalents of formic acid to a solution of iridium precatalyst **1** leads to formation of a new species (intermediate A) with a hydride signal at -19.43 ppm (Supplementary Figures 7-8). Data for this species is consistent with oxidative addition of formic acid to the iridium precatalyst. 20 minutes after addition of sodium formate and formic acid, a time course experiment was collected over a period of 66 min (Supplementary Figure 8). The resulting stacked ^{1}H NMR spectra shows that intermediate A grows then intermediate B appears ca. 30 min after addition of sodium formate and formic acid. Intermediate B is consistent with a species where one of the cyclooctadiene double bonds is bound to iridium and the other is free. Then the final product (a Pfaltz-type dimer) appears ca. 1 hour after addition. The ^{1}H NMR spectrum of the final product is consistent with dimer **2** (Supplementary Figure 9; S = CD_3CN). The cyclooctadiene in the iridium precatalyst is reduced to cyclooctene and free cyclooctene is seen in the ^{1}H NMR spectrum of the products. Although **3** does not form in CD_3CN, it is reasonable to conclude that formation of the catalyst resting state (diformate **3a**) proceeds in a similar fashion through the intermediacy of **2** when formic acid or tetraglyme is used as solvent. It is worth noting that, in CD_3CN, iridium **1** reacts in the absence of base with formic acid to form **2**, but in a much slower rate.

The formation of Pfaltz-type dimer **2** is also observed upon reaction of precatalyst **1** with hydrogen. Supplementary Figure 10 shows the Pfaltz type dimer and cyclooctene formed from this reaction in CD_3CN solvent. Cyclooctene is slowly hydrogenated when the reaction mixture is allowed to sit at room temperature.

*Observation of Intermediate **3** in Formic Acid*

We studied the catalyst resting state(s) in formic acid by dissolving, in the drybox, 10.0 mg of the iridium precatalyst and 10.0 mg of sodium formate in 1.0 mL of proteo formic acid in a J-Young tube. The J-Young tube was taken out of the drybox and was connected to a three-way

valve, which was connected to a nitrogen line and a 50.0 mL gas burette. The tubing and the gas burette were purged with nitrogen for ca. 15 minutes. The plug valve and the three-way valve were opened such that gas produced go directly to the gas burette. The reaction flask was heated in an oil bath to 70 ^{o}C. About 15 mL of gas was produced during heating. The solution was then allowed to cool to room temperature. The proteo formic acid solvent was evaporated under high vacuum and the resulting residue was left under high vacuum overnight. The residue was then dissolved in formic acid-d_2. The ^{1}H NMR spectrum of the residue is shown in Supplementary Figure 11. Examination of the hydride region reveals two resting states: (A) a minor resting state with two different hydrides at -27.24 and -28.51 ppm that integrate in a 2:1 ratio (consistent with the hydrides in the ^{1}H NMR of **3b**, whose X-ray structure is known). We formulate this species as dimer **3a**. (B) The major resting state contains three different hydrides at -19.41, -25.05, and -27.13 ppm. We formulate this species as dimer **4** (see Figure 4).

Proton-Hydride Fidelity Experiment

In the drybox, a J-Young tube was charged with the iridium precatalyst (1.8 mg, 2.64 μmol), sodium formate (1.7 mg, 25.0 μmol), formic acid-d_1 (O-D; 5 μL, 0.13 mmol), and 0.6 mL of methanol-d_4. A ^{1}H NMR time course experiment spanning over 72 minutes was performed at 70 °C. Before the time course experiment was begun, the NMR tube had been heated ca. 10 minutes at 70 °C and much H-D had formed (Supplementary Figure 12). Nevertheless, formation of predominantly H-D with a small amount of H_2 is observed. This observation is consistent with a formic acid dehydrogenation mechanism that proceeds through formation of an iridium monohydride where the hydride comes from the formyl C-H bond of formic acid. This iridium monohydride is then protonated (or, technically, deuterated) by the formic acid O-D. We expect that an iridium dihydride that undergoes reductive elimination to yield H_2 should enable scrambling of proton and hydride, thus we disfavor this possibility. The small amount of H_2 that forms can be rationalized by the presence of small amounts of formic acid O-H bonds which protonates the iridium monohydride. Interestingly, the HD signal disappears after extended heating, which is consistent with a slow reverse reaction when the reaction is run in a sealed vessel. Such back reaction is impossible under our kinetics acquisition conditions because the H_2 product is sequestered and quantified in an eudiometer.

Supplementary Methods

General Experimental Information

All air and water sensitive procedures were carried out either in a Vacuum Atmosphere glove box under nitrogen (2-10 ppm O_2 for all manipulations) or using standard Schlenk techniques under nitrogen. Dichloromethane-d_2, acetonitrile-d_3, methanol-d_4, water-d_2, formic acid-d_1 (C–D), and formic acid-d_1 (O–D) NMR solvents and reagents were purchased from Cambridge Isotopes Laboratories. Formic acid-d_2 was purchased from SynQuest Laboratories. Dichloromethane, ethyl ether, and hexanes were purchased from VWR and dried in a J. C. Meyer solvent purification system with alumina/copper(II) oxide columns; chloro(1,5-cyclooctadiene)iridium(I) dimer (Strem), sodium trifluoromethanesulfonate (Sigma-Aldrich), and formic acid (Sigma-Aldrich) were purchased and used as received; 2-((di-t-butylphosphino)methyl)pyridine was synthesized using a literature procedure.[18]

NMR spectra were recorded on a Varian VNMRS 500 or VNMRS 600 spectrometer. All chemical shifts are reported in units of ppm and referenced to the residual ^{1}H or ^{13}C solvent peak and line-listed according to (s) singlet, (bs) broad singlet, (d) doublet, (t) triplet, (dd) double doublet, etc. ^{13}C spectra are delimited by carbon peaks, not carbon count. ^{31}P chemical shifts are referenced to an 85% phosphoric acid external standard. Air-sensitive NMR spectra were taken in 8" J-Young tubes (Wilmad or Norell) with Teflon valve plugs. MALDI mass spectra were obtained on an Applied Biosystems Voyager spectrometer using the evaporated drop method on a coated 96 well plate. The matrix was 2,5-dihydroxybenzoic acid. In a standard preparation, ca. 1 mg analyte and ca. 10 mg matrix was dissolved in methanol and spotted on the plate with a glass capillary. X-ray crystallography data were obtained on a Bruker APEX DUO single-crystal diffractometer equipped with an APEX2 CCD detector, Mo fine-focus and Cu micro-focus X-ray sources. Elemental analysis data were obtained on a Thermo Flash 2000 CHNS Elemental Analyzer. Gas chromatography data were obtained on a Thermo gas chromatograph (Supelco Carboxen®-1010 plot, 30 m × 0.53 mm) equipped with a TCD detector (detection limit: 0.099 v/v %). IR data (1 cm^{-1} resolution) were obtained on a Bruker Vertex 80v FT-IR using a deuterated triglycine sulfate (DTGS) detector.

Characterization of Complex 1 and Complex 3b

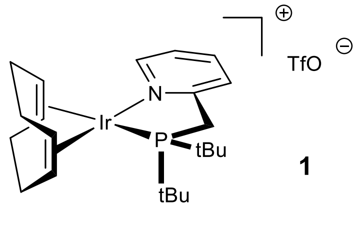

1

^{1}H NMR (500 MHz, methylene chloride-d_2) δ 8.27 (d, J = 4.5 Hz, 1H), 8.10 (t, J = 7.5 Hz, 1H), 8.02 (d, J = 7.5 Hz, 1H), 7.47 (d, J = 5.5 Hz, 1H), 4.89 (s, 2H), 4.51 (s, 2H), 3.66 (d, J = 9.1 Hz, 2H), 2.39 (m, 2H), 2.33-2.17 (m, 4H), 1.96 (m, 2H), 1.34 (d, J = 13.9 Hz, 18H).

^{13}C NMR (126 MHz, methylene chloride-d_2) δ 169.60 (d, J = 20.2 Hz), 149.37, 141.94, 125.25 (d, J = 8.8 Hz), 124.74, 90.70, 90.61, 63.70, 37.64 (d, J = 20.2 Hz), 34.79 (d, J = 25.2 Hz), 33.57, 33.55, 30.27, 30.25, 28.41, 28.40.

^{31}P NMR (202 MHz, methylene chloride-d_2) δ 57.98.

^{19}F NMR (470 MHz, methylene chloride-d_2) δ -78.91.

Elemental Analysis (CHNS) Anal. Calcd for $C_{23}H_{36}F_3IrNO_3PS$: C, 40.22; H, 5.28; N, 2.04; S, 4.67. Found: C, 40.54; H, 5.40; N, 2.01; S, 4.63.

IR (thin film/cm^{-1}) ν 2954, 2889, 2836, 1608, 1475, 1389, 1370, 1319, 1273, 1223, 1148, 1108, 1080, 1031, 1001, 967, 894, 875, 821, 776, 636.

MS (MALDI) calc'd for $[C_{23}H_{36}F_3IrNO_3PS]^+$ 687.2 g/mol, found 687.0 g/mol.

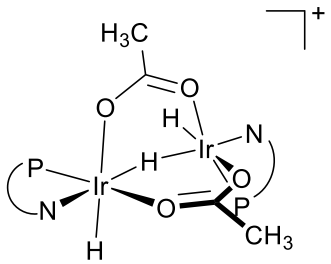

3b

^{1}H NMR (600 MHz, methylene chloride-d_2): δ = 9.23 (dd, J = 5.9, 1.6 Hz, py 2H), 7.93 (tt, J = 7.7, 1.4 Hz, py 2H), 7.70 (d, J = 7.9 Hz, py 2H), 7.36 (ddd, J = 7.4, 5.8, 1.5 Hz, py 2H), 3.58 (dd, J = 16.7, 9.6 Hz, methylene 2H), 3.43 (dd, J = 16.7, 10.9 Hz, methylene 2H), 2.07 (d, J = 1.6 Hz, μ-acetate 6H) 1.25 (d, J = 14.0 Hz, tBu 18H), 1.19 (d, J = 13.9 Hz, tBu 18H), -26.30 (dd, J = 19.0, 3.1 Hz, Ir-*H* 2H), -28.76 (m, μ-*H* 1H).

^{13}C NMR (150 MHz , methylene chloride-d_2): δ = 184.63 (acetate), 163.84 (py), 149.59 (py), 138.94 (py), 123.42 (py), 123.16 (py), 36.78 (d, J = 30.0 Hz, P-*C*), 36.38 (d, J = 27.5 Hz, P-*C*), 34.88 (d, J = 26.9 Hz, P-*C*), 29.79 (d, J = 25.6 Hz, P-*C*), 28.95 (tBu methyl), 28.69 (tBu methyl), 24.27 (acetate methyl). Other small peaks on the spectrum appeared overtime due to the instability of this compound in solution.

^{31}P NMR (243 MHz, methylene chloride-d_2): δ = 44.97.

^{19}F NMR (564 MHz, methylene chloride-d_2): δ = -79.43.

IR (thin film/cm^{-1}) ν 3584, 3441, 2956, 2917, 2238, 2078, 1589, 1479, 1433, 1392, 1371, 1264, 1224, 1158, 1031, 822, 770, 638.

MS (MALDI) calc'd for $[C_{30}H_{53}Ir_2N_2O_4P_2]^+$ 951.3 g/mol, found 951.1 g/mol.

Experiments that Support Homogeneous Catalysis

Visual Appearance

The reaction mixture is a translucent pale yellow solution during catalysis, with no dark precipitates forming. At the end of the reaction, a pale orange solid (the catalysts) remains.

Well Behaved Kinetics

The reaction follows well-behaved saturation catalysis kinetics through its pseudo-zero order region, which persists until solvent level drops appreciably as solvent is consumed (Supplementary Figure 1).

Mercury Drop Test

The mercury drop test was performed by placing 0.5 mL of a formic acid stock solution with 0.01 mol% in iridium precatalyst **1** and 5 mol% sodium formate into a 5 mL reaction flask possessing a large bore plug valve and a side arm. A drop of mercury was then added to this solution. The rate of formic acid decomposition (average of two runs) was then measured to be 1.24×10^{-7} mol s^{-1} at 90 °C, which is just slightly slower compared to the measured rate of formic acid decomposition in the absence of mercury (1.35×10^{-7} mol s^{-1}).

Quantitative Poisoning

Phenanthroline was utilized as catalytic poison. 1.00 mL (1.26 μmol iridium) of a formic acid solution that is 1.26 mM in iridium atom and 1.26 M in sodium formate was placed in a 2 mL volumetric flask. One-half mole equivalent of the poison relative to the iridium catalyst was added as a stock solution in formic acid (100 μL of 6.3 mM solution, 0.63 μmol). The solution was diluted to 2.00 mL. Using 0.5 mL of this solution, the rate of formic acid decomposition (average of two runs) was measured to be 1.27×10^{-8} mol s^{-1} at 86 °C, which is 62% of the formic acid decomposition rate in the absence of phenanthroline (which was measured as 2.04×10^{-8} mol s^{-1}).

Kinetic Isotope Studies

To obtain values for k_H/k_D, the initial rates of dehydrogenation using formic acid, formic acid-d_2, formic acid-d_1 (O–D), and formic acid-d_1 (C–D) were obtained.

Stock solutions of formic acid, formic acid-d_2, formic acid-d_1 (C-D), and formic acid-d_1 (O-D) that are 0.005 mol% (50 ppm) in iridium precatalyst **1** and 5 mol% in formate were prepared. In the drybox, a vial was charged with 1.8 mg (2.6 μmol) of the iridium precatalyst and 180 mg of either sodium formate (for the FA and FA-d_1 (O-D) solutions) or sodium formate-d_1 (for the FA-d_2 and FA-d_1 (C-D) solutions). The appropriate proteo/deutero formic acid (2.0 mL) was then added to dissolve the base and the precatalyst. The solution was allowed to sit overnight before use. The initial rates of formic acid decomposition were obtained using Procedure 2 (see Methods). See main text for the obtained kinetic isotope effects.

Supplementary References

1. Bielinski, E. A. *et al.* Lewis acid-assisted formic acid dehydrogenation using a pincer-supported iron catalyst. *J. Am. Chem. Soc.* **136**, 10234-10237 (2014).
2. Boddien, A., *et al.* Efficient Dehydrogenation of formic acid using an iron catalyst. *Science* **333**, 1733-1736 (2011).A. Boddien *et al.*, *Science* **333**, 1733 (2011).
3. DeRight, R. E. The decomposition of formic acid by sulfuric acid. *J. Am. Chem. Soc.* **55,** 4761-4764 (1933).
4. Yu, J. & Savage, P. E. Decomposition of formic acid under hydrothermal conditions. *Ind. Eng. Chem. Res.* **37**, 2-10 (1998).
5. Akiya, N. & Savage, P. E. Role of water in formic acid decomposition. *AIChE J.* **44**, 405-415 (1998).
6. Boddien, A., Loges, B., Junge, H., & Beller, M. Hydrogen generation at ambient conditions: application in fuel cells. *ChemSusChem* **1**, 751-758 (2008).
7. Hull, J. F. *et al.* Reversible hydrogen storage using CO_2 and a proton-switchable iridium catalyst in aqueous media under mild temperatures and pressures. *Nat. Chem.* **4**, 383-388 (2012).
8. Bertini, F., Mellone, I., Ienco, A., Peruzzini, M., & Gonsalvi, L. Iron(II) complexes of the linear *rac*-tetraphos-1 ligand as efficient homogeneous catalysts for sodium bicarbonate hydrogenation and formic acid dehydrogenation. *ACS Catal*. **5**, 1254-1265 (2015).
9. Zell, T., Butscke, B., Ben-David, Y., & Milstein, D. Efficient hydrogen liberation from formic acid catalyzed by a well-defined iron pincer complex under mild conditions. *Chem. Eur. J.* **19**, 8068-8072 (2013).
10. Manaka, Y. *et al.* Efficient H_2 generation from formic acid usine azole complexes in water. *Catal. Sci. Technol.* **4**, 34-37 (2014).
11. Barnard, J. H., Wang, C., Berry, N. G., & Xiao, J. Long-range metal-ligand bifunctional catalysis: cyclometallated iridium catalysts for the mild and rapid dehydrogenation of formic acid. *Chem. Sci.* **4**, 1234-1244 (2013).
12. Himeda, Y. Highly efficient hydrogen evolution by decomposition of formic acid using an iridium catalyst with 4,4'-dihydroxy-2,2'-bipyridine. *Green Chem.* **11**, 2018-2022 (2009).
13. Oldenhof, S., *et al.* Base-free production of H_2 by dehydrogenation of formic acid using an iridium-bisMETAMORPhos complex. *Chem. Eur. J.* **19**, 11507-11511 (2013).
14. Myers, T. W., & Berben, L. A. Aluminum-ligand cooperation promotes selective dehydrogenation of formic acid to H_2 and CO_2. *Chem. Sci.* **5**, 2771-2777 (2014).
15. Fukuzumi, S. Kobayashi, T., & Suenobu, T. Unusually large tunneling effect on highly efficient generation of hydrogen and hydrogen isotopes in pH-selective decomposition of formic acid catalyzed by a heterodinuclear iridium–ruthenium complex in water. *J. Am. Chem. Soc.* **132**, 1496-1497 (2010).
16. Fellay, C., Dyson, P. J., & Laurenczy, G. A viable hydrogen-storage system based on selective formic acid decomposition with a ruthenium catalyst. *Angew. Chem. Int. Ed.* **47**, 3966-3968 (2008).
17. Morris, D. J., Clarkson, G. J., & Wills, M. Insights into hydrogen generation from formic acid using ruthenium complexes. *Organometallics* **28**, 4133-4140 (2009).

18. Beddie, C., Wei, P., & Douglas, S. Titanium pyridyl-phosphinimide complexes Synthesis, structure, and ethylene polymerization catalysis. *Can. J. Chem.* **84**, 755-761 (2006).
